# Long‐Term Salt Exposure Reprograms the *Nicotiana tabacum* BY‐2 Suspension Proteome and Metabolome Toward Stabilization of the Core Metabolic Pathways, Protein Turnover Machinery Modifications, and Protective Metabolome Adjustments

**DOI:** 10.1111/pce.70581

**Published:** 2026-05-05

**Authors:** Anita Rzadkiewicz, Łukasz Marczak, Aleksander Strugała, Maria Tomys, Ewelina Ratajczak, Tomasz Skrzypczak, Przemysław Wojtaszek, Anna Kasprowicz‐Maluśki, Agnieszka Szuba

**Affiliations:** ^1^ Polish Academy of Sciences Institute of Dendrology Kórnik Greater Poland Poland; ^2^ Institute of Bioorganic Chemistry Polish Academy of Sciences Poznań Greater Poland Poland; ^3^ Faculty of Biology Adam Mickiewicz University Poznań Greater Poland Poland; ^4^ Centre of Advanced Technologies Adam Mickiewicz University Poznań Greater Poland Poland

**Keywords:** β‐sitosterol, adaptation to salt, new homeostasis state, osmoadaptation, osmoprotectants, post‐transcriptional regulation, proline, protein turnover, ribosomes, RNA‐binding proteins

## Abstract

In this study, we analyzed a unique *Nicotiana tabacum* BY‐2 line that was gradually adapted to and subsequently maintained in 190 mM NaCl for over 15 years. Years of continuous high salinity shaped a stable “new homeostasis” in BY‐2 suspension cells. Salt‐adapted cells were smaller and formed tighter clusters. Metabolomics revealed constitutive enrichment of osmoprotectants and antioxidant‐associated metabolites—including proline, GABA, and selected purine derivatives—and a marked increase in β‐sitosterol, together pointing to osmoadaptation, membrane stabilization, and ROS buffering without broad induction of classical antioxidant enzymes. Proteomics showed modest changes dominated by information‐processing layers: higher abundance of histone deacetylases, RNA‐binding and splicing‐related factors, and enzymes linked to mRNA polyadenylation/decapping. In contrast, many 60S ribosomal proteins were less abundant, indicating restrained translation. Despite persistent osmotic pressure, enzymes of central metabolism changed little overall, whereas lipid‐associated shifts and sterol enrichment suggest ongoing membrane remodeling. Collectively, these multi‐omics data indicate that long‐term salt adaptation in BY‐2 cells prioritizes small‐molecule osmolytes and post‐transcriptional control over costly protein turnover, supporting sustained function under high osmolarity. This work provides novel insights into the molecular features underlying the reprogramming of the metabolome and proteome, enabling plant cells to survive long‐term in high‐salinity conditions.

## Summary statement

Long‐term salt‐adapted *Nicotiana tabacum* BY‐2 cells are smaller and accumulate osmoprotectants and antioxidant metabolites, but not anti‐ROS enzymes or HSPs. Ribosomal proteins are reduced, proteins linked to alternative splicing and mRNA stability regulation increase, whereas central metabolic proteins remain relatively stable.

## 1 Introduction

A large share of plant habitats is affected by salinity and osmotic stress, which impair cell function and survival (Richter et al. 2019; Horie et al. 2011), and are expected to increasingly limit organismal performance. At the same time, the molecular basis of long‐term adaptation to salt and osmotic stress conditions remains unclear (Szuba et al. 2022; Skrzypczak et al. 2025; Tibesigwa et al. 2025). Metabolic responses depend on the duration of stress (Schwachtje et al. 2019). Therefore, we expect that the adapted cells develop specific protective mechanisms distinct from those observed during acute or even chronic stress responses.

Short‐term salt stress typically triggers bursts of reactive oxygen species (ROS), leading to DNA and protein damage, as well as attempts at their repair (Zhang and Sonnewald 2017; Szuba and Lorenc‐Plucińska 2018; Tibesigwa et al. 2025). To protect macromolecules, plant cells activate Heat Shock Proteins (HSPs) overproduction and ROS scavenging, with increased antioxidants and antioxidative enzymes being a universal stress response (Szuba and Lorenc‐Plucińska 2018; Zhang et al. 2020; Tibesigwa et al. 2025). A key feature of osmotic (including salt) stress response is overproduction of small, water‐soluble osmoprotectants—proteins, amino acids, amines, betaines (e.g., glycine betaine), organic acids, sugars, or polyols. Some of these compounds, such as proline, buffer redox state via ROS scavenging, increase intracellular pressure to prevent plasmolysis, and stabilize membranes and proteins (Pontecorvo et al. 2011). Short‐ and long‐term (over a week) osmotic stress disrupts key physiological processes in plants, including hormonal metabolism, respiration, and water relations (Pontecorvo et al. 2011). Salt stress dysregulates central pathways—glycolysis, the Krebs cycle, mitochondrial electron transport, and photosystems, leading to impaired energy conversion and altered biosynthesis of TCA cycle intermediates, which are essential for growth and production of osmoprotectants and antioxidants, among others (Król and Weidner 2017; Zhang and Sonnewald 2017). Response to harsh environments is costly, as resources are diverted to attempts to restore homeostasis, protect or repair cellular components, and reprogram metabolism (Nadarajah 2020). Growth inhibition and reduced plant size are commonly observed under both acute and chronic stress (Pontecorvo et al. 2011; Szuba et al. 2022). Comparative studies of stress‐sensitive and ‐tolerant ecotypes reveal that tolerant plants, developed over generations of plants living in unfavorable conditions, exhibit greater resilience and more stable molecular stress responses, with key pathways less perturbed than in sensitive ecotypes (Kovalchuk et al. 2004; Sairam et al. 2005). Similarly, time‐course experiments and studies on acclimated plants demonstrate that long‐term tolerance involves increased molecular stability and protection of macromolecules, particularly proteins (Huang et al. 2014; Szuba and Lorenc‐Plucińska 2018). Nevertheless, the “new homeostasis state” commonly postulated for adapted plants requires detailed molecular characterization (Cappetta et al. 2025), especially in systems capable of surviving many years under constant stress.

High salinity persistently forces plant cells to increase cytosolic density to avoid plasmolysis, as observed also in the salt‐adapted BY‐2 cell line used in this study (Skrzypczak et al. 2025), raising the question of how long‐term intra‐ and extracellular pressure impacts the function of key cell (macro)molecules, like proteins or nucleic acids.

Proteins are essential for cell growth and physiology, and their biosynthesis is one of the most energy‐ and nutrient‐demanding cellular processes (Kafri et al. 2016). Under acute stress, translation can consume up to 50% of cellular energy to produce protective proteins such as HSPs, anti‐ROS enzymes, or osmoprotective proteins (Nadarajah 2020; Cappetta et al. 2025). In marine organisms adapted to high osmotic pressure, proteins maintain enzymatic activity through subtle structural modifications, and pressure‐resistant isoforms have been observed (Ohmae et al. 2015; Scepankova et al. 2023). However, the main and direct mechanism regulating the functioning and turnover of proteins is the regulation of their biosynthesis and degradation, primarily through the function of ribosomes and/or 26S proteasomes (Ingvardsen and Veierskov 2001; Komili et al. 2007; Szuba and Lorenc‐Plucińska 2018). Cytoplasmic co‐solutes, such as proline, which are typically upregulated in response to salt stress, generally stabilize proteins (Auton et al. 2011; Schwinefus et al. 2017). Prolonged protein longevity benefits cells by reducing protein turnover costs (Ingvardsen and Veierskov 2001; Szuba and Lorenc‐Plucińska 2018). If osmolytes are found to be upregulated in salt‐adapted BY‐2 cells, then we expect to observe symptoms of protein turnover stabilization.

Unlike proteins, osmolytes tend to destabilize nucleic acids, particularly RNA (Lambert and Draper 2007; Schwinefus et al. 2017), which probably necessitates the adjustments in nucleic acid metabolism in salt‐adapted plant cells. The RNA fate and activity are primarily regulated by RNA‐binding proteins (RBPs), whose roles under salinity are largely unknown but are generally considered to be predominantly positive (Rosa Téllez et al. 2020). Transcription factors are typically upregulated under salt stress to induce stress‐tolerant genes, but their activity depends on DNA accessibility, modulated, for example, by epigenetic regulations (Fernando 2020; Wang et al. 2024). Stress also increases the frequency and diversity of alternative splicing events, a crucial plant adaptive mechanism (Rosa Téllez et al. 2020; Verta and Jacobs 2022; Alhabsi et al. 2025). Enzymes regulating mRNA polyadenylation adjust stress‐related protein synthesis by controlling transcript stability, while regulating mRNA degradation rates further contributes to stress responses (Zhao et al. 2009; Kawa and Testerink 2017). Altogether, these RNA‐related processes are expected to contribute significantly to the molecular balance of salt‐adapted BY‐2 cells.

We therefore hypothesize that long‐term salinity establishes a “new homeostasis” supported by (H1) constitutive accumulation of osmolytes, (H2) compensatory adjustments in RNA metabolism and gene expression machinery, and (H3) restrained translation and protein turnover. To test these hypotheses, we performed a proteome–metabolome analysis of *Nicotiana tabacum* BY‐2 cells that were gradually salt‐adapted and maintained for years under high osmolarity. This system offers a uniform genetic background, controlled cultivation, and continuous selection over hundreds of cell generations, enabling the dissection of molecular mechanisms underlying long‐term salt adaptation.

## 2 Materials and Methods

### 2.1 Plant Material

*Nicotiana tabacum* BY‐2 suspension cells were cultured in modified Murashige and Skoog medium at 22°C, 120 rpm, in darkness (Skrzypczak et al. 2025) (Supplementary File S1). Over an 18‐month period, BY‐2 cells were gradually adapted to 190 mM NaCl, with concentrations increasing every 2 months from an initial 20 mM. The final osmolarity of fresh media was approximately 550 mOsm kg^−1^ for the adapted line and 220 mOsm kg^−1^ for controls. After initial gradual adaptation, both control and salt‐adapted BY‐2 cells were subcultured weekly in their respective media for over 15 years. All experiments were conducted on BY‐2 cells maintained under these final osmotic conditions for approximately 860 subcultures (Supplementary File S1). Before experiments, synchronization was improved by leaving the cultures unpassaged for 2 weeks, followed by adding 5 mL of the previous culture to 70 mL of new medium, exactly as described previously (Skrzypczak et al. 2025). Undifferentiated, proliferative cells were detected 2 days post‐subculture, whereas differentiated, non‐proliferative cells were observed after 10 days of culture. For analyses, sampling was performed on the third day of the logarithmic growth phase of the analyzed BY‐2 cells.

### 2.2 Microscopic Study *–* Plant Cell Size Estimation

To visualize cell size and shape, BY‐2 cells were labeled with 5 μM Di‐4‐ANEPPDHQ membrane dye (Merck) in appropriate culture medium for 5 min, washed with culture medium, and imaged with a Leica SP8 confocal microscope. The sample was excited using a 488‐nm laser, and the detection was set to the 504–577 nm range. A 63× water immersion objective (N.A. = 1.2) was used. Images were analyzed with ImageJ software (NIH, USA).

### 2.3 Biochemical Study

For biochemical and omics analyses, cells were drained using Miracloth (Merck), immediately frozen in liquid nitrogen, and then ground with a cryomill. The resulting BY‐2 powder was stored at –80°C until use.

*Total nitrogen and carbon* were determined in ground and dried samples (*n* = 5) with a CHNS analyzer (2400 CHNS/O Series II System, PerkinElmer, Waltham, MA, USA), after which the C/N ratio was calculated.

*Soluble nonstructural carbohydrates* (SCs) and starch were extracted from dried suspension cells following a standard colorimetric assay (Szuba et al. 2013). SC polysaccharides were hydrolyzed with sulfuric acid, converted to monosaccharides, and quantified via anthrone reaction at 625 nm using glucose as a standard. Residual starch was hydrolyzed with α‐amylase–glucosidase, detected with oxidase–peroxidase/o‐dianisidine dihydrochloride at 450 nm, and likewise standardized to glucose. SC and starch contents (*n* = 6) were normalized to the fresh weight of BY‐2 cells.

*The total soluble phenolics* in BY‐2 cells were determined by a colorimetric assay (Johnson and Schaal 1957). Phenolics were extracted with ethanol from dried cell powder (*n* = 6), reacted with Folin–Ciocalteu reagent in Na₂CO₃, and quantified at 660 nm. Results were expressed as µmol chlorogenic acid (CGA) g⁻¹ fresh weight (FW).

*Abscisic acid (ABA)* was quantified using an ELISA kit (Agrisera AS20 4392, with a detection range of 0.156–10 μgmL^−1^) following the manufacturer's protocol. Frozen BY‐2 cell powder (100 mg, *n* = 5) was extracted in 50 µL Agrisera extract buffer, and supernatants were subjected to ELISA. HRP‐conjugated antibody was visualized with TMB, and absorbance was measured at 450 nm. ABA standards were used for calibration, and results were expressed as µgg⁻¹ FW.

*Proline* was quantified following a modified method (Carillo et al. 2011). Frozen BY‐2 cell powder (70 mg, *n* = 4) was extracted overnight in ethanol: water (40:60, v/v). Supernatants were reacted with 1% ninhydrin in 60% acetic acid/20% ethanol, and absorbance was measured at 520 nm. Proline concentrations (µMg⁻¹ FW) were determined using a proline standard curve.

*The H*_*2*_ *O*_*2*_ content was determined according to Zhou et al. (2006). BY‐2 cell powder (250 mg, *n* = 6) was extracted at 4°C in activated carbon and 5% TCA (1:0.2:1 w/w/v). H₂O₂ was quantified colorimetrically using 0.6 mM 4‐(2‐pyridylazo) resorcinol disodium salt with 0.6 mM titanium potassium oxalate (1:1) in 50 mM phosphate buffer (pH 8.4), measured at 508 nm, and normalized to a H₂O₂ standard curve. Results were expressed as μmolg⁻¹ FW.

*Lipid peroxidation* was estimated via malondialdehyde (MDA) content. Frozen BY‐2 cells (*n* = 6) were homogenized in 0.25 M H₂SO₄/10% TCA (Shah et al. 2001). Thiobarbituric acid (0.25% v/v) was added to visualize MDA, and supernatants were measured at 532 nm (reaction products) and 600 nm (nonspecific absorbance). MDA levels were expressed per g FW.

*Total antioxidant capacity* (TAC) was measured using a colorimetric kit (Sigma‐Aldrich MAK187). Frozen BY‐2 cell powder (20 mg, *n* = 5) was extracted in 50 mM sodium phosphate buffer (pH 7.0) with 0.2 mM EDTA, 2% PVPP, and 2 mM PMSF, and analyzed per the manufacturer's instructions. Absorbance at 570 nm was used to calculate TAC, expressed as µmol Trolox equivalents g⁻¹ FW.

### 2.4 GC‐MS Metabolome Analysis

Metabolites were extracted from 100 mg BY‐2 cells (*n* = 5) using methanol (Szuba et al. 2020) and analyzed on a Thermo TSQ 8000 triple‐quadrupole mass spectrometer connected to a TRACE 1300 gas chromatograph. Mass spectra were recorded from 35 to 850 m/z and normalized to the total ion chromatogram (TIC) peak areas. Data were processed in Perseus v2.0.11.0 (all detected compounds) and MetaboAnalyst 6.0 (compounds assigned to molecular formulas) for statistical analysis. Detailed GC‐MS protocol and additional data are provided in Supplementary File S2 and untransformed metabolome data have been deposited in the open Zenodo repository (https://doi.org/10.5281/zenodo.19219373).

### 2.5 Proteomic Analysis

Total proteins were extracted from 500 mg BY‐2 cells (*n* = 5) using a modified phenolic method (Szuba et al. 2020). Protein concentrations were determined with the 2D Quant Kit (GE Healthcare).

General protein profiles were analyzed by 1D electrophoresis (Szuba et al. 2013) (20 µg per lane) and visualized with Coomassie Brilliant Blue or used for Western blotting (see Supplementary File S3).

*For MS/MS analysis*, protein extracts, quality‐checked by 1D electrophoresis (Supplementary File S3), were standardly digested in solution (10 µg per run) and analyzed by nano‐UPLC‐MS/MS on a Thermo Exploris 480 q‐orbitrap mass spectrometer. For each run, 5 µL of peptide solution was used. The raw data were processed with FragPipe v22.0 using the MSFragger search engine (fragpipe.nesvilab.org/) against the UniProt database with *Nicotiana tabacum* taxonomy filter. Data were evaluated in Perseus v2.0.11.0, excluding reverse identifications (decoy analysis), contaminants, and proteins identified only by modification sites. Additionally, in order to verify the abundance of selected enzymes of the core metabolism, Parallel Reaction Monitoring (PRM) analysis was performed, a highly sensitive and selective method enabling quantitative determination of specific proteins (Bourmaud et al. 2016). LC‐MS/MS additional data, including PRM protocols and results, are provided in Supplementary File S4. Untransformed proteome data have been deposited in the open Zenodo repository (https://doi.org/10.5281/zenodo.19219373). To determine the levels of transcripts of these selected enzymes, an analysis of mRNA levels was carried out using the RT‐qPCR method. Detailed protocol and results are provided in Supplementary File S5.

### 2.6 Statistics and Data Analysis

Biochemical and biometric data were considered statistically significant at *p* < 0.05, as determined by *t*‐tests in JMP Pro 13.0.0 (SAS Institute). MS/MS data were log‐transformed, blanks removed, and missing values imputed from a normal distribution as needed. Processed matrices were used for *t*‐tests, PCA, volcano plots, and hierarchical clustering. Metabolites and proteins with FDR ≤ 0.05 and fold change (FC) ≥ 2 were considered significant; those with 1.1 ≤ FC < 2 are provided in Supplementary Files S2 and S4. Hierarchical clustering data were Z‐score normalized, and only proteins that showed significant changes are shown in the heatmaps.

## 3 Results

**Figure 1:**
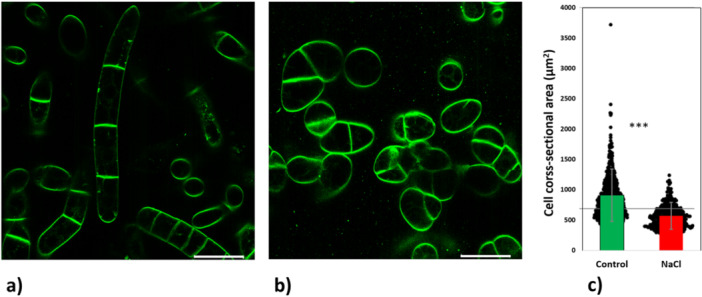
Microscopic analysis of analyzed BY‐2 cells. Representative image of control (a) and adapted to 190 mM NaCl (b) BY‐2 suspensions. Scale bar = 50 µm. Cross‐sectional area of control and adapted BY‐2 cells (*n* ≥ 400) (c). Mean ± SD values, along with the distribution of measurements for all analyzed BY‐2 cells, are presented in the graph; significant difference was assessed according to the *t*‐test. ***p < 0.001.

Aside from isolated suspension cells, control BY‐2 cells often appeared as strings of single elongated cells connected only by “cross walls,” whereas adapted cells formed multicellular, more compact clusters (Figure 1a vs. b). BY‐2 cells adapted to salt stress were approximately half the size of control cells (Figure 1c).

**Figure 2:**
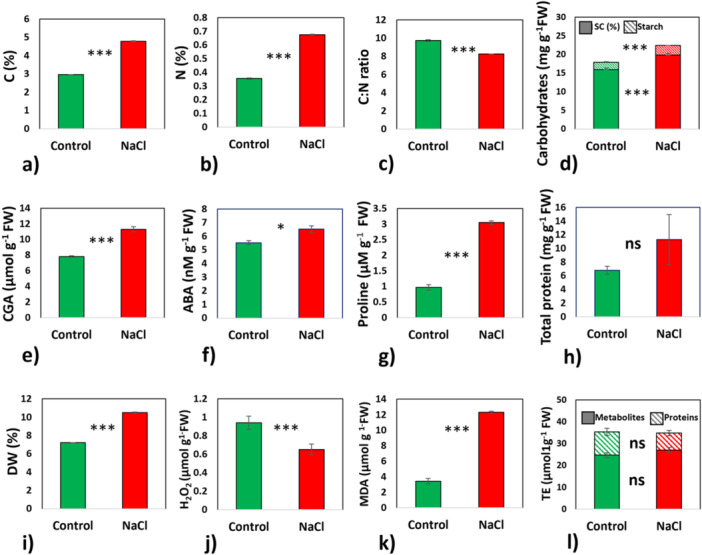
Basic biochemistry of BY‐2 cells. Measurements for control BY‐2 suspension cell line (control) and adapted to NaCl line (NaCl). Carbon (a) and nitrogen (b) percentage and C:N ratio (c) (*n* = 5). Non‐structural carbohydrates, including soluble non‐structural carbohydrates (SC) and starch concentrations, were calculated against glucose as a standard (*n* = 6) (d). Total phenolic compounds concentrations (normalized against chlorogenic acid (CGA) as a standard (*n* = 6) (e). ABA concentrations (*n* = 5) (f). Proline concentrations (*n* = 5) (g). Total protein concentrations representing protein extracted for proteome analysis (h). Dry matter percentage (i). Hydrogen peroxide (H_2_O_2_) (j) and malondialdehyde (MDA) concentrations (*n* = 6) (k). Total antioxidant capacity (TAC), including activity of the low molecular mass compounds (metabolites) and enzymatic activity (proteins; *n* = 5) (l). The values are expressed as the means ± SEs. Significant differences (*p* ≤ 0.05) were assessed according to a *t*‐test. FW, fresh weight; *p < 0.05; ***p < 0.001; ns, not significant. All values (except (i)) were normalized by the fresh weight of BY‐2 suspension cells. [Color figure can be viewed at wileyonlinelibrary.com]

Cells maintained under constant high salt exhibited significantly increased carbon (Figure 2a) and nitrogen (Figure 2b) contents, with a greater relative increase in nitrogen, resulting in a decreased C:N ratio in salt‐adapted cells (Figure 2c).

Salt‐adapted cells exhibited increased levels of soluble non‐structural carbohydrates and starch, with SCs dominating the total non‐structural carbohydrate pool (Figure 2d). Total phenolics (Figure 2e), ABA (Figure 2f), and, especially, proline (Figure 2g) were also elevated. Total protein content, assessed via extraction efficiency, tended to be higher (Figure 2h). All these increases coincided with higher dry matter content in salt‐adapted cells compared to controls (Figure 2i).

Level of H₂O₂, the most abundant ROS in plant cells, was lower in salt‐adapted cells (Figure 2j), whereas lipid peroxidation was nearly threefold higher under NaCl (Figure 2k). Total antioxidant capacity (TAC) did not differ between control and salt‐adapted cells, considering both low‐molecular‐weight antioxidants and enzymatic contributions (Figure 2l). A non‐significant trend toward higher relative contribution of low‐molecular‐weight antioxidants and reduced protein‐based TAC was observed in salt‐adapted cells (Figure 2l).

### 3.1 BY‐2 Cells Metabolomic Adaptations

**Figure 3:**
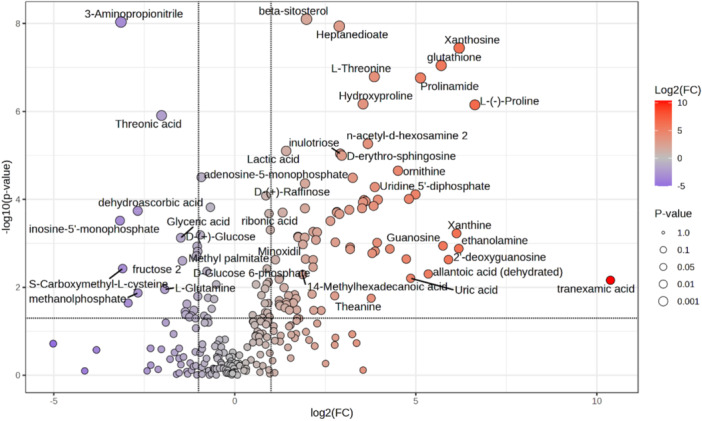
Volcano plot representing differentially abundant metabolites. Differentially abundant metabolites (*p* < 0.05; FC ≥ 2.0) identified in the molecular structure were subjected to analysis. Red points indicate more abundance in salt‐adapted cells, blue points indicate those more abundant in control cells. For the full list of differentially abundant compounds, please see Supplementary File S2. [Color figure can be viewed at wileyonlinelibrary.com]

Adaptation to NaCl caused significant changes in the metabolome of BY‐2 suspension cells (Supplementary Figure S2‐1 and S2‐2). The majority of differential metabolites selected on the basis of a *t*‐test (117 from 272 total identified ones) were more abundant in salt‐adapted cells (Figure 3 and Supplementary Figure S2‐3, and Supplementary File S2), confirming our hypothesis (H1).

Consistent with elevated total nitrogen in salt‐adapted cells (Figure 2b), many highly enriched metabolites were N‐compounds, including numerous amino acids and derivatives such as threonine, hydroxyproline, and proline, the latter showing one of the highest fold changes (Figure 3 and Supplementary Figure S2‐3, and Supplementary File S2). Enriched metabolites also included purines and purine nucleosides, for example, xanthosine, xanthine, guanosine, and 2′‐deoxyguanosine (Figure 3). Among other enriched compounds, glutathione increased, whereas dehydroascorbic acid decreased in the salt‐adapted BY‐2 line, indicating a shift toward a more reduced redox state, consistent with sustained antioxidant protection in long‐term salt‐adapted cells.

**Figure 4:**
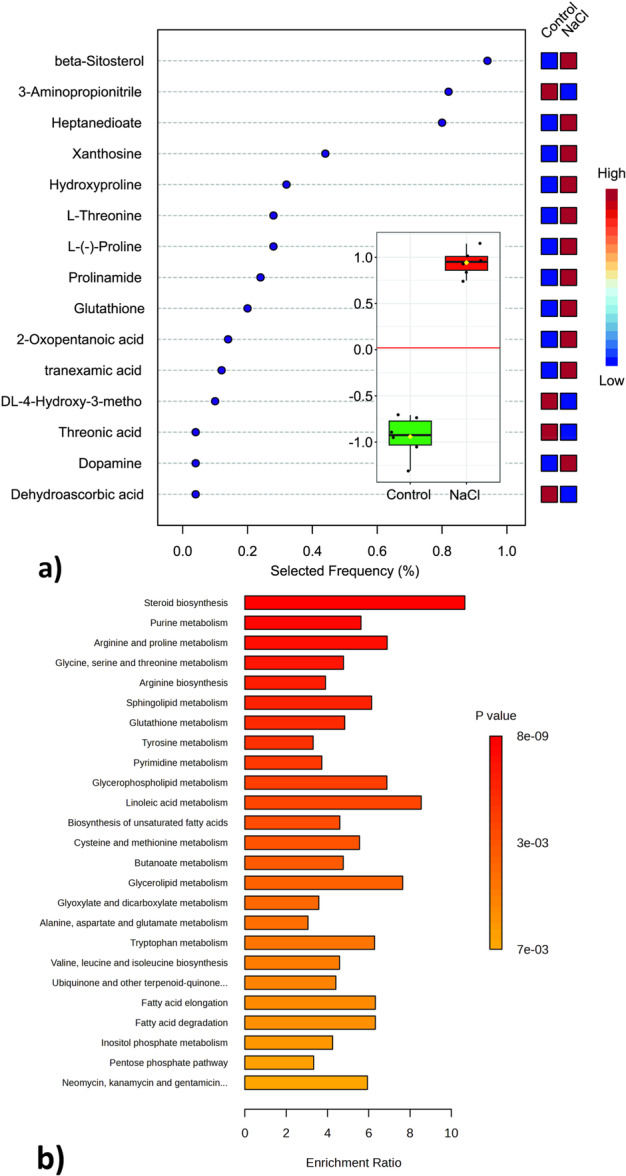
Metabolome analysis. (a) Vip metabolites. Frequency plot representing the top 15 dysregulated metabolites according to the classical univariate receiver operating characteristic (ROC) analysis utilizing PLS‐DA as the classification method and PLS‐DA built‐in as the feature ranking method. The ROC analysis revealed that beta‐sitosterol was selected as the most promising biomarker of salt‐stress adaptation, with an AUC (area under the ROC curve) of 1. The box plot representing the relative abundance of beta‐sitosterol is also presented. (b) Quantitative enrichment of metabolites (25 top‐most enriched pathways are presented). [Color figure can be viewed at wileyonlinelibrary.com]

Among the most altered metabolites, β‐sitosterol was identified, followed by heptanedioate, both of which were more abundant in salt‐adapted cells (Figure 4a). Additionally, 3‐aminopropionitrile was found to be one of the few significantly less abundant compounds in salt‐adapted cells (Figures 3 and 4). Few carbohydrates differed between treatments—both higher (maltose and raffinose) and lower (fructose and glucose) levels were observed in salt‐adapted cells (Figure 3 and Supplementary File S2). Increased compounds also included γ‐aminobutyric acid (GABA) and lactic acid. Long‐chain fatty acids (oleic, linoleic, and palmitic acids) and esters decreased under salt stress, whereas fatty acyl glycosides of mono‐ and disaccharides (palatinose and turanose) increased (Figure 3 and Supplementary File S2). Several key glycolysis and TCA cycle intermediates showed slight decreases with relatively low fold changes (Supplementary File S2).

Enrichment of β‐sitosterol (and its stearate) along with lipid‐related metabolites aligned with the results of molecular pathway enrichment, based on the abundance of all identified metabolites. This indicates alterations in processes related to membrane function, including steroid biosynthesis, sphingolipid metabolism, and fatty acid metabolism. However, the most affected pathways were those associated with amino acid metabolism. Changes in nucleotide levels were reflected in the enrichment of purine and pyrimidine metabolism pathways (Figure 4b). Overall, the metabolomic profile of salt‐adapted BY‐2 cells suggests a reprogramming toward osmolyte accumulation, membrane reinforcement, and sustained antioxidant defense, while maintaining core metabolic stability.

### 3.2 Modification of the Proteome in Salt‐Adapted BY‐2 Suspension Cells

High‐throughput LC‐MS/MS identified 1964 proteins, with only 74 showing differential abundance between control and salt‐adapted cells, including 54 with FC ≥ 2. Most of these were more abundant in the adapted variant. These results were consistent with 1D electrophoresis, which showed no major changes in the proteome profile of the salt‐adapted cells (Supplementary File S3).

#### 3.2.1 Anti‐Oxidative Response

**Figure 5:**
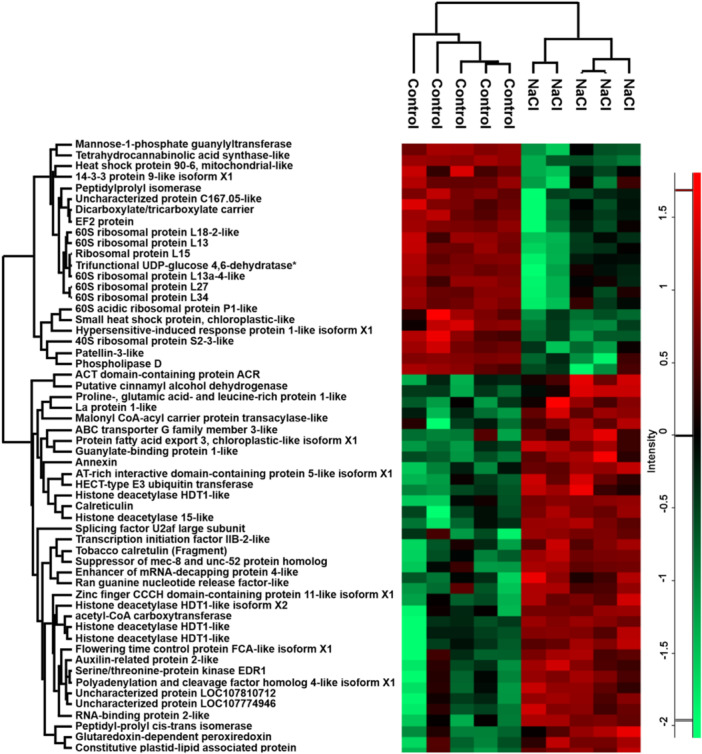
Protein heatmap. Heat map analysis combined with hierarchical cluster analysis reveals differentially abundant proteins in BY‐2 suspension cells (*p* < 0.05; FC ≥ 2.0) according to *t*‐tests (control vs. salt‐adapted (NaCl) variants). Green, minimal abundance; red, maximal abundance. [Color figure can be viewed at wileyonlinelibrary.com]

Only a few ROS‐related enzymes were differentially abundant between control and salt‐adapted BY‐2 cells. Mannose‐1‐phosphate guanylyltransferase, a key enzyme in ascorbate biosynthesis (Kumar et al. 2012), was less plentiful, whereas glutaredoxin‐dependent peroxiredoxin was more abundant in salt‐adapted cells. Organelle HSPs and the mitochondrial organization–associated hypersensitive‐induced response protein 1‐like isoform X1 were decreased in salt‐adapted cells (Figure 5).

#### 3.2.2 Signal Transduction and Cellular Trafficking

In salt‐adapted cells, several regulatory proteins were altered. 14‐3‐3 and phospholipase D were less abundant, whereas nucleic acid‐binding proteins, serine/threonine kinases, and Ran guanine nucleotide release factor–like proteins were more abundant. Patellin, a putative membrane trafficking protein (Zhou et al. 2018), and dicarboxylate/tricarboxylate carrier, involved in mitochondrial membrane transport, were less abundant, but ACT domain‐containing protein ACR, engaged inter alia in solute transport, and ABC transporter, a multi‐pass membrane protein, were more abundant in salt‐adapted cells (Figure 5).

#### 3.2.3 Chromatin State, Transcription Factors, and mRNA Maturation and Decay

Guanylate‐binding protein 1‐like, involved in stress‐triggered regulation of the nucleus liquid–liquid phase separation (Huang et al. 2021), was more abundant in salt‐adapted cells. Histone deacetylases, responsible for histone modifications, were elevated compared to controls. Transcription initiation factor IIB‐2‐like, which also contributes to alternative splicing (Miranda‐Ríos et al. 2021), along with splicing‐related proteins (U2af large subunit and suppressor of mec‐8 and unc‐52 homolog), were increased (Figure 5). Transcription elongation factors were decreased. In contrast, La‐protein, involved in tRNA production and translation regulation, was upregulated in salt‐adapted BY‐2 cells. All differentially abundant enzymes involved in mRNA polyadenylation and decapping were more abundant in salt‐adapted cells. Finally, zinc finger CCCH domain–containing protein 11‐like, which is involved in nuclear mRNA export (Chen et al. 2020), was upregulated in salt‐adapted cells (Figure 5).

#### 3.2.4 Ribosomes and Protein Maturation

Ribosomal proteins, particularly those in the 60S large subunit, were the most numerous among proteins decreased in salt‐adapted BY‐2 cells, representing ~19% of all differentially abundant proteins (Supplementary File S4). This aligns with the high ribosome content characteristic of BY‐2 cells in the logarithmic growth phase. All differentially abundant ribosomal proteins were reduced in salt‐adapted cells (Figure 5 and Supplementary File S4). In contrast, proline‐, glutamic acid‐, and leucine‐rich protein 1‐like, involved in rRNA transcription and in a key step of ribosome biogenesis (Gonugunta et al. 2011), were increased. Actin cytoskeleton–regulating annexin, a calcium‐dependent phospholipid‐binding protein involved in clathrin‐coated vesicle regulation and endo/exocytosis, was upregulated, as was auxilin‐related protein 2, a co‐chaperone in clathrin‐mediated endocytosis (Adamowski et al. 2018). ER chaperones calreticulin and calretulin were also more abundant. Peptidyl‐prolyl isomerases exhibited mixed changes, with both increases and decreases observed. Only one protein degradation‐related enzyme differed between control and adapted cells: HECT‐type E3 ubiquitin transferase, which was more abundant in salt‐adapted cells. Reduced ribosomal protein abundance suggests restrained translation activity, consistent with an energy‐saving strategy during long‐term stress (H3).

#### 3.2.5 Lipids

Proteins more abundant in salt‐adapted cells included enzymes involved in long‐chain fatty acid biosynthesis and mitochondrial precursor transport (malonyl‐CoA‐acyl carrier protein transacylase‐like, acetyl‐CoA carboxytransferase). Fatty acid transporters and storage proteins (protein fatty acid export 3, chloroplastic‐like isoform X1, and constitutive plastid‐lipid–associated protein) were also more abundant in adapted BY‐2 cells (Figure 5).

#### 3.2.6 Cell Wall

Proteomic analysis indicated cell wall modulation, with upregulation of putative cinnamyl alcohol dehydrogenase, involved in the final step of monolignol biosynthesis, and downregulation of trifunctional UDP‐glucose 4,6‐dehydratase, which regulates primary cell wall rhamnogalacturonans.

Interestingly, no representatives of the core metabolic processes were detected among the variable proteins. To further confirm this assumption, we thoroughly checked the levels of selected key enzymes involved in the most important core metabolic process—glycolysis, using alternative western blot and PRM techniques (Supplementary Files S3 and S4). We also checked the levels of the transcripts encoding them (Supplementary File S5). Our results confirmed stable levels of the analyzed proteins and their encoding mRNA.

The full list of differentially abundant proteins is provided in Supplementary File S4, and gel‐related data are provided in Supplementary File S3.

**Figure 6:**
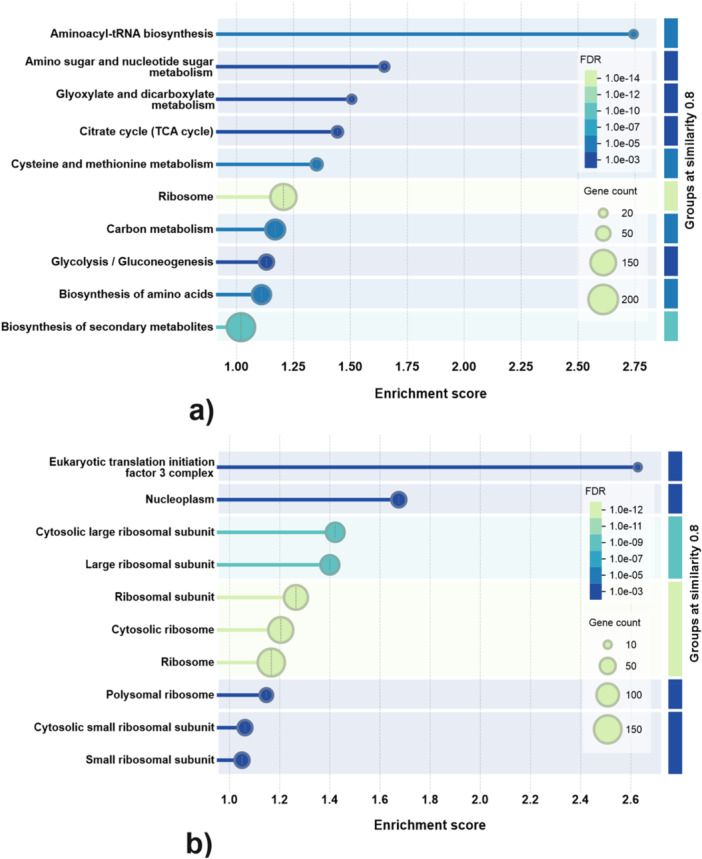
Functional enrichment analysis. KEGG pathway enrichment (a) and subcellular localization (compartments) enrichment (b). Analysis performed using the STRING v. 12.0 platform using all identified proteins (see Supplementary File S4 for the complete protein list) and log2FC as the input values. The top 10 enriched categories are shown; full results are provided in Supplementary File S4. [Color figure can be viewed at wileyonlinelibrary.com]

**Figure 7:**
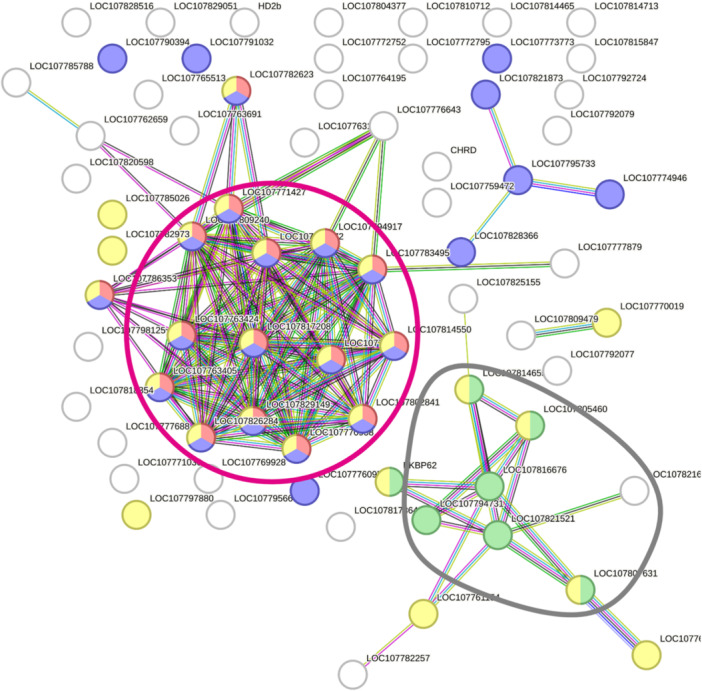
Protein–protein interaction network (prepared using STRING v. 12.0 platform). Differentially abundant proteins (identified via *t*‐test in Perseus software) were used for this analysis under multiple proteins mode. Nodes represent proteins, edges indicate interactions, and edge color saturation corresponds to confidence scores ( ≥ 0.4). The node degree reflects the average number of interactions per node, while the clustering coefficient indicates the network density. Node colors represent molecular pathway enrichment: violet (gene expression); red (translation), green (protein folding), and yellow (protein metabolic processes). The KEGG pathways were marked by cycles: pink (ribosome) and gray (proteomic processing in the endoplasmic reticulum). For LOC‐numbers explanation, see Supplementary File S4. [Color figure can be viewed at wileyonlinelibrary.com]

Differentially abundant proteins, especially those with FC ≥ 2 (Figure 5), only partially explained KEGG pathway enrichment, which was analyzed based on all identified proteins' abundance changes (Figure 6). This highlights the important role of less pronounced changes in protein levels. The most enriched pathway was aminoacyl‐tRNA biosynthesis, related to ribosome function, followed by nucleotide metabolism (Figure 6a). Amino acid biosynthesis and modification were also markedly affected, along with the TCA cycle and glycolysis/gluconeogenesis. Enrichment in carbon metabolism mainly involved glyoxylate and dicarboxylate pathways (Figure 6a), even though no individual enzymes in these core pathways, like in other central metabolic processes, met the *t*‐test significance thresholds (FC ≥ 2; *p* < 0.05; Figure 7, Supplementary File S4). The dominance of ribosome‐related proteins among those with the most significant abundance changes (Figure 5) was reflected in subcellular localization enrichment, which was dominated by ribosomal compartments (Figure 6b).

The predominance of ribosomal subcellular localization among differentially abundant proteins (Figure 6b) was also evident in protein–protein interaction analysis. Network analysis revealed that proteins with the highest number of interactions (Figure 7) were primarily involved in gene expression, translation, and protein metabolism, all of which are related to ribosome‐associated KEGG pathways. Proteins involved in protein folding formed a second key network, representing ER‐based protein maturation processes. Another smaller network included proteins related to gene expression (Figure 7).

Together, proteomic data suggest that long‐term salt adaptation stabilizes the BY‐2 proteome by restraining translation, reinforcing membranes, and enhancing RNA processing and protein‐folding systems.

## 4 Discussion

### 4.1 Plant Cell Adjustments to Long‐Term Life in a Surrounding With High Osmolarity

Long‐term exposure to high salinity forces plant cells to strike a balance between maintaining structural integrity, adjusting to osmotic stress, and conserving energy.

In plants that have been adapted for many years, survival has been achieved primarily through reduced size, coexisted with cytoplasmic densification under osmotic stress (Bandehagh and Taylor 2020; Colin et al. 2022). Our study revealed that in years‐adapted plant cells, survivability was achieved by sacrificing plant cell size, as previously suggested (Zhang et al. 2020). Salt‐adapted BY‐2 cells mitigate negative salt influence also via enhanced clustering, promoted by extracellular polysaccharides (Santos et al. 2016; Kasprowicz‐Maluśki, unpublished data), which correlates with increased lactic acid levels (Banti et al. 2013). Salt‐adapted cells also exhibit proteomic signals of cell wall modulation, a crucial adaptive trait (Colin et al. 2022).

The most pronounced differences between control and salt‐adapted BY‐2 cells were observed at the metabolome level, primarily reflecting short‐term osmotic responses (Schwachtje et al. 2019), but they seem to have become constitutive over prolonged adaptation, indicating the establishment of a new homeostatic state (H1). Major adjustments included increases in osmoprotectants such as amino acids—tryptophan, proline, and GABA (Krasensky and Jonak 2012; Ghosh et al. 2022; Dabravolski and Isayenkov 2023)—confirming our hypothesis H1. The metabolome adjustments largely mirrored short‐term osmotic responses (Schwachtje et al. 2019). However, in contrast to stress response (Ghosh et al. 2022; Szuba et al. 2022; Dong et al. 2024), no proteomic evidence was found indicating intensified biosynthesis or degradation of proline or any other differentially abundant metabolite in salt‐adapted BY‐2, suggesting that the high level of osmoprotectants is maintained constitutively under prolonged salt exposure, rather than through active, ongoing enzymatic regulation. These changes, similar to those seen in salt‐tolerant halophytes (Wang et al. 2016), successfully enhanced cytoplasmic density and molecular crowding in adapted BY‐2 cells (Skrzypczak et al. 2025), preventing plasmolysis, among other effects. This observation supports the concept of “metabolic pre‐adaptation,” where protective metabolites remain continuously elevated without acute transcriptional and translational activation (Chun et al. 2019).

Co‐solutes, such as xanthine and proline, may also act as antioxidants, chelating and buffering ROS (Meena et al. 2019; Sun et al. 2021). Maintaining ROS homeostasis is crucial for salt tolerance (Cao et al. 2023). Unlike short‐term stress, where many antioxidative enzymes are upregulated (e.g., Kim et al. 2022), ROS buffering in salt‐adapted BY‐2 cells appears independent, mainly of anti‐ROS enzymes, with only glutaredoxin‐dependent peroxiredoxin being increased (Wang et al. 2020). This is supported by decreased patellin‐3‐like protein, which is involved in cellular ion and redox homeostasis and is usually upregulated under oxidative stress (Zhou et al. 2018). Such remodeling suggests that salt‐adapted cells maintain redox balance primarily through constitutive metabolite pools rather than enzyme induction.

At the same time, the BY‐2 cell membrane was constantly exposed to high ion concentrations, as indicated by increased MDA levels in salt‐exposed BY‐2 cells. The need for membrane protection was reflected by a significant increase in β‐sitosterol, a known biomarker of salt adaptation and stress tolerance (Li et al. 2019). Membrane sensitivity and its increased turnover were further suggested by changes in lipid metabolism, with decreased fatty acids coexisting with an increased abundance of enzymes involved in their biosynthesis and transport. It should be noted that lipids, besides serving as membrane building blocks, are also a significant source of energy, undergoing β‐oxidation in mitochondria (Allenbach and Poirier 2000). Although slight decreases in central intermediates, such as pyruvate and succinate, were observed, no significant changes in the TCA cycle or glycolysis—commonly seen under short‐term salt stress (Athar et al. 2022; Szuba et al. 2022)—were detected, supporting the idea that cells maintain a new homeostatic state. The lack of changes in key metabolic enzymes, as well as in their transcript levels, suggests a high degree of cellular homeostasis in core metabolism in long‐term adapted plant cells, particularly given the frequent discrepancies between protein and transcript levels reported in plants (Lan et al. 2012; Aslam et al. 2024; Vélez‐Bermúdez and Schmidt 2014).

The substantial increase in heptanedioate, a dicarboxylic acid involved in biotin synthesis (Tawaraya et al. 2014), and also a compensatory product of fatty acid ω‐oxidation (Allenbach and Poirier 2000; Miura 2013), nevertheless suggests potential activation of alternative energy pathways.

### 4.2 Signals of Adjustments in Alternative Splicing, and mRNA Stability

Metabolome adjustments that prevent plasmolysis and oxidative stress in salt‐adapted BY‐2 cells increase cytoplasmic density and molecular crowding (Skrzypczak et al. 2025), affecting major cellular processes in largely unknown ways.

Beyond metabolic adjustments, osmotic adaptation in BY‐2 cells involved profound modifications of RNA metabolism and gene expression machinery. Osmotic stress typically modulates chromatin structure and nuclear–cytoplasmic trafficking to enhance nucleocytoplasmic transport (Finan et al. 2011). In adapted BY‐2 cells, nuclear mRNA export, previously linked to improved drought tolerance via the ABA signaling pathway (Chen et al. 2020), was likely also enhanced, as shown inter alia by increased levels of ABA, zinc finger CCCH domain–containing protein 11‐like or guanylate‐binding protein 1‐like (GBPL1) (Honkala et al. 2020; Tang et al. 2022).

The pronounced upregulation of histone deacetylases (HDACs) in salt‐adapted BY‐2 cells is linked to adaptation through epigenetic regulation of gene expression (Kumar et al. 2021; Wang et al. 2024). The lack of changes in histone acetyltransferases, however, suggests a shift toward reduced DNA accessibility through histone N‐tail modification, as it is known that histone acetylation relaxes chromatin structure, increasing DNA accessibility to transcription factors (Wang et al. 2024). Despite the increased abundance of transcription factors in salt‐adapted BY‐2 cells, commonly observed also during short‐term salt exposure (Fernando 2020), we detected additional evidence supporting the aforementioned hypothesis. Namely, GBPL1, more abundant in adapted cells, may act as an allosteric inhibitor limiting chromatin access for other GTPases (Huang et al. 2021), while AT‐rich interactive domain‐containing protein 5‐like isoform X1, a DNA‐binding protein, functions as a transcriptional repressor. HDACs may also regulate stress adaptation by deacetylating not only histones but also transcription factors, signaling components, and ribosomal proteins (Cui et al. 2023), all of which exhibit altered abundance in BY‐2 adapted cells.

Chromatin remodeling influences alternative splicing (AS) under osmotic stress (Yu et al. 2019). It was demonstrated that HDT15 is upregulated in salt‐adapted BY‐2 cells and may regulate intron retention of ABA‐responsive genes, a process that ABA itself enhances (Tu et al. 2022). The crucial role of AS in long‐term salt stress adaptation (Rosa Téllez et al. 2020; Verta and Jacobs 2022; Alhabsi et al. 2025) was supported here by the detection of many differentially abundant AS‐related proteins—primarily increased in salt‐adapted BY2‐2 cells. Changes in numerous AS‐associated proteins emphasize the key role of this process in plant cells that have endured generations of salt exposure, but also highlight the uniqueness of long‐term adaptation state. Our results indicate that in years‐long adapted BY‐2 cells, AS likely modulates the transcriptome and translation machinery itself, rather than stress‐responsive genes, as observed in various stressed plants (Yao et al. 2024; Cappetta et al. 2025). This is further supported by the detection of AS‐related proteins, including serine/arginine‐rich (SR) proteins and transcription factor II B, both of which are essential for polyadenylation and pre‐mRNA splicing and are themselves subject to AS to enhance salt tolerance in planta (Schimanski 2006; Gu et al. 2020).

In salt‐adapted BY‐2 cells, we observed higher levels of the auxiliary spliceosomal protein suppressor of mec‐8 and unc‐52 homolog, which regulates alternative splice site selection in a small set of pre‐mRNA targets (Chung et al. 2009). This up‐regulation suggests that in long‐term adapted plant cells, AS target specificity is critical. Alternative splicing may also drive the accumulation of primary salt‐response metabolites through adaptation‐triggered variation, for example, in proline metabolism (Kesari et al. 2012).

Numerous RNA‐binding proteins were found to be differently abundant between the analyzed variants. Among them, multifunctional RBPs, such as RBP2, which regulate alternative splicing, mRNA stability, translation, and RNA editing (Quilloy and Reyes 2024), as well as mitochondrial oxidative stress (Kim et al. 2007), were upregulated. RNA chaperones involved in tRNA production and mRNA translation, such as La protein 1‐like, also prevent protein misfolding (Dock‐Bregeon et al. 2021) and are more abundant, highlighting the key role of RNA and protein‐RNA interactions in long‐term survival in a high osmotic environment.

Modulation of mRNA longevity likely represents the next key adaptive mechanism in plant cells, as indicated by the increased abundance of enzymes regulating polyadenylation (Zhao et al. 2009) and transcript degradation (Kawa and Testerink 2017). Our results align with recent studies indicating that plant adaptation to salt stress may involve changes in pre‐mRNA processing mechanisms (Zhang et al. 2026).

In salt‐adapted BY‐2 cells, the Enhancer of mRNA‐decapping protein 4 is more abundant and associated with TOR‐regulated pathways involved in energy, translation, transcription, and lipid biosynthesis (Wu et al. 2020), processes that are extensively remodeled in these cells. Degradation of stress‐induced transcripts is crucial for stress recovery, thereby restoring baseline mRNA levels (Kawa and Testerink 2017). Adjustments in highly substrate‐specific decapping proteins (Belostotsky and Sieburth 2009) suggest that this process is also critical for establishing and maintaining a new homeostatic state in adapted plants.

Together, these changes, consistent with hypothesis H2, indicate that RNA metabolism is not merely a stress response but a major pillar of the long‐term adaptive state in salt‐adapted plant cells—a mitigation of osmolyte‐induced RNA destabilization (Lambert and Draper 2007).

### 4.3 Adjustments in Ribosomes, Protein Maturation, and Protein Degradation in Salt‐Adapted Cells

At the proteome level, the most prominent signal of long‐term adaptation was the reduced abundance of 60S ribosomal proteins, alongside increases in multifunctional proteins potentially involved in ribosome function, such as Proline‐, Glutamic acid‐, and Leucine‐rich protein 1‐like (Gonugunta et al. 2011) or HDT1, which regulates ribosome biogenesis and heterogeneity (Luo et al. 2022). Variations in ribosomal composition or relative protein abundance may form a “ribosome code” that drives ribosome specialization rather than loss of translational capacity (Komili et al. 2007), a process important in adapted cells. Changes in ribosome protein abundances likely reflect, therefore, energy‐saving adjustments in protein synthesis, consistent with our hypothesis of reduced protein turnover (H3). Supporting this, elongation factors such as Elongation Factor 2 and 60S acidic ribosomal protein P1 (Shi et al. 2019; Siodmak et al. 2023) were less abundant in salt‐adapted BY‐2 cells.

The lack of increase in the abundance of enzymes associated with protein degradation indicates an unchanged level of protein degradation processes. This suggests that, unlike short‐term stress (Lyzenga and Stone 2012; Szuba et al. 2022), long‐term salt exposure did not result in enhanced intracellular protein damage, as further evidenced by the lack of severe nitrotyrosine level increase—a protein oxidative stress fingerprint (Corpas et al. 2013)—in the salt‐adapted BY‐2 proteome. In fact, the abundance of Hypersensitive‐Induced Response Protein 1‐like isoform X1 decreased, suggesting reduced protein degradation activity (Zhou et al. 2010). A single selective substrate‐recruiting enzyme of the ubiquitin/26S proteasome system (UPS) was more abundant. It is therefore tempting to conclude that in long‐term adapted cells, protein degradation may be based on selectivity rather than intensity (Al‐Saharin et al. 2022).

Our results suggest that long‐term salt adaptation minimizes protein damage and turnover costs, indicating a transition from reactive defense to proteome stabilization. Indeed, in contrast to RNAs, co‐solute accumulation stabilizes proteins (Auton et al. 2011). Highly elevated proline in salt‐adapted BY‐2 cells acted as a molecular chaperone, protecting enzyme integrity and activity (Meena et al. 2019; Dong et al. 2024). This may explain the absence of HSPs induction—a typical short‐term stress response (Szuba and Lorenc‐Plucińska 2018; Szuba et al. 2022)—suggesting that multifunctional metabolites, rather than proteins, fulfill this protective role in long‐term adaptation. Peptidylprolyl isomerases, mediating protein assembly and maturation, enhance osmotic stress tolerance (Sharma and Singh 2003; Singh et al. 2021) and showed varied adjustments in salt‐adapted cells. The critical role of protein maturation, as detected in our study, including the key role of the ER network, may reflect the need for specific protein structural modifications to adapt to elevated intercellular pressure (Ohmae et al. 2015; Scepankova et al. 2023). Overall, these results suggest that the adapted proteome achieves energetic efficiency through restrained translation, selective degradation, and enhanced folding quality.

## 5 Conclusions

In smaller, salt‐adapted BY‐2 cells, we observed signals of reduction of energy‐consuming protein turnover to the necessary minimum, accompanied by essentially unchanged levels of core metabolism, consistent with the establishment and maintenance of a new homeostatic state. No significant proteomic adjustments were detected in energy production machinery, suggesting that reduced cell size likely reflects a metabolic shift from growth toward enhanced biosynthesis of low‐molecular‐weight, multifunctional osmoprotectants, which also act as successful antioxidants. Concurrently, characteristic adjustments were observed in the transcription and protein turnover machinery itself, including alternative splicing, mRNA stability (with a focus on polyadenylation and decapping), and ribosome regulation, which were predominant without an upregulation of canonical (oxidative) stress‐response proteins. Overall, our study provides unique insights into the molecular features of long‐term salt‐adapted plant cells.

## Ethics Statement

The authors have nothing to report.

## Consent

The authors have nothing to report.

## Conflicts of Interest

The authors declare no conflicts of interest.

## Policy on Using ChatGPT and Similar AI Tools

AI tools (DeepL, Grammarly, and ChatGPT‐5) were used for linguistic corrections.

## Supporting information

Supporting File 1

Supporting File 2

Supporting File 3

Supporting File 4

Supporting File 5

## Data Availability

The primary data generated or analyzed during this study are included in this published article and its Supporting Files. Additional datasets used and/or analyzed during this study are available from the corresponding author upon reasonable request. Anna Kasprowicz‐Maluśki maintains the BY‐2 lines used in this study at the Faculty of Biology, Adam Mickiewicz University, Poznań, Poland.

## References

[pce70581-bib-0001] Adamowski, M. , M. Narasimhan , U. Kania , M. Glanc , G. De Jaeger , and J. Friml . 2018. “A Functional Study of AUXILIN‐LIKE1 and 2, Two Putative Clathrin Uncoating Factors in *Arabidopsis* .” Plant Cell 30, no. 3: 700–716. 10.1105/tpc.17.00785. 29511054 PMC5894831

[pce70581-bib-0002] Alhabsi, A. , Y. Ling , M. Crespi , A. S. N. Reddy , and M. Mahfouz . 2025. “Alternative Splicing Dynamics in Plant Adaptive Responses to Stress.” Annual Review of Plant Biology 76, no. 1: 687–717. 10.1146/annurev-arplant-083123-090055. 39952682

[pce70581-bib-0003] Allenbach, L. , and Y. Poirier . 2000. “Analysis of the Alternative Pathways for the β‐Oxidation of Unsaturated Fatty Acids Using Transgenic Plants Synthesizing Polyhydroxyalkanoates in Peroxisomes.” Plant Physiology 124, no. 3: 1159–1168. 10.1104/pp.124.3.1159. 11080293 PMC59215

[pce70581-bib-0004] Al‐Saharin, R. , H. Hellmann , and S. Mooney . 2022. “Plant E3 Ligases and Their Role in Abiotic Stress Response.” Cells 11, no. 5: 890. 10.3390/cells11050890. 35269512 PMC8909703

[pce70581-bib-0005] Aslam, N. , Q. Li , S. Bashir , L. Yuan , L. Qiao , and W. Li . 2024. “Integrated Review of Transcriptomic and Proteomic Studies to Understand Molecular Mechanisms of Rice's Response to Environmental Stresses.” Biology 13, no. 9: 659. 10.3390/biology13090659. 39336087 PMC11428526

[pce70581-bib-0006] Athar, H. R. , F. Zulfiqar , A. Moosa , et al. 2022. “Salt Stress Proteins in Plants: An Overview.” Frontiers in Plant Science 13: 999058. 10.3389/fpls.2022.999058. 36589054 PMC9800898

[pce70581-bib-0007] Auton, M. , J. Rösgen , M. Sinev , L. M. F. Holthauzen , and D. W. Bolen . 2011. “Osmolyte Effects on Protein Stability and Solubility: A Balancing Act Between Backbone and Side‐Chains.” Biophysical Chemistry 159, no. 1: 90–99. 10.1016/j.bpc.2011.05.012. 21683504 PMC3166983

[pce70581-bib-0008] Bandehagh, A. , and N. L. Taylor . 2020. “Can Alternative Metabolic Pathways and Shunts Overcome Salinity Induced Inhibition of Central Carbon Metabolism in Crops?” Frontiers in Plant Science 11: 1072. 32849676 10.3389/fpls.2020.01072 PMC7417600

[pce70581-bib-0009] Banti, V. , B. Giuntoli , S. Gonzali , et al. 2013. “Low Oxygen Response Mechanisms in Green Organisms.” International Journal of Molecular Sciences 14, no. 3: 4734–4761. 10.3390/ijms14034734. 23446868 PMC3634410

[pce70581-bib-0010] Belostotsky, D. A. , and L. E. Sieburth . 2009. “Kill the Messenger: mRNA Decay and Plant Development.” Current Opinion in Plant Biology 12, no. 1: 96–102. 10.1016/j.pbi.2008.09.003. 18990607

[pce70581-bib-0011] Bourmaud, A. , S. Gallien , and B. Domon . 2016. “Parallel Reaction Monitoring Using Quadrupole‐Orbitrap Mass Spectrometer: Principle and Applications.” Proteomics 16, no. 15–16: 2146–2159. 10.1002/pmic.201500543. 27145088

[pce70581-bib-0012] Cao, D. , W. Zhang , and N. Yang , et al. 2023. “Proteomic and Metabolomic Analyses Uncover Integrative Mechanisms in *Sesuvium portulacastrum* Tolerance to Salt Stress.” Frontiers in Plant Science 14: 1277762. 10.3389/fpls.2023.1277762. 38089796 PMC10714944

[pce70581-bib-0013] Cappetta, E. , C. Del Regno , S. Ceccacci , et al. 2025. “Proteome Reprogramming and Acquired Stress Tolerance in Potato Cells Exposed to Acute or Stepwise Water Deficit.” Plant Cell & Environment 48, no. 5: 2875–2894. 10.1111/pce.15306. PMC11963495 39639630

[pce70581-bib-0014] Carillo, P. , and Y. Gibon , Contributors P . 2011. “PROTOCOL: Extraction and Determination of Proline.” Prometheus 2011: 1–5. www.researchgate.net.

[pce70581-bib-0015] Chen, F. , H.‐L. Liu , K. Wang , Y.‐M. Gao , M. Wu , and Y. Xiang . 2020. “Identification of CCCH Zinc Finger Proteins Family in Moso Bamboo (*Phyllostachys edulis*), and PeC3H74 Confers Drought Tolerance to Transgenic Plants.” Frontiers in Plant Science 11: 579255. 10.3389/fpls.2020.579255. 33240298 PMC7680867

[pce70581-bib-0016] Chun, H. J. , D. Baek , H. M. Cho , et al. 2019. “Metabolic Adjustment of Arabidopsis Root Suspension Cells During Adaptation to Salt Stress and Mitotic Stress Memory.” Plant and Cell Physiology 60, no. 3: 612–625. 10.1093/pcp/pcy231. 30496500

[pce70581-bib-0017] Chung, T. , D. Wang , C.‐S. Kim , R. Yadegari , and B. A. Larkins . 2009. “Plant SMU‐1 and SMU‐2 Homologues Regulate Pre‐mRNA Splicing and Multiple Aspects of Development.” Plant Physiology 151, no. 3: 1498–1512. 10.1104/pp.109.141705. 19734266 PMC2773069

[pce70581-bib-0018] Colin, L. , F. Ruhnow , J.‐K. Zhu , C. Zhao , Y. Zhao , and S. Persson . 2022. “The Cell Biology of Primary Cell Walls During Salt Stress.” Plant Cell 35: 201–217. 10.1093/plcell/koac292. PMC9806596 36149287

[pce70581-bib-0019] Corpas, F. J. , J. M. Palma , L. A. del Río , and J. B. Barroso . 2013. “Protein Tyrosine Nitration in Higher Plants Grown Under Natural and Stress Conditions.” Frontiers in Plant Science 4: 2013. 10.3389/fpls.2013.00029. PMC3580390 23444154

[pce70581-bib-0020] Cui, X. , A. Dard , J.‐P. Reichheld , and D.‐X. Zhou . 2023. “Multifaceted Functions of Histone Deacetylases in Stress Response.” Trends in Plant Science 28, no. 11: 1245–1256. 10.1016/j.tplants.2023.06.006. 37394308

[pce70581-bib-0021] Dabravolski, S. A. , and S. V. Isayenkov . 2023. “The Role of the γ‐Aminobutyric Acid (GABA) in Plant Salt Stress Tolerance.” Horticulturae 9, no. 2: 230. 10.3390/plants14142162.

[pce70581-bib-0022] Dock‐Bregeon, A. C. , K. A. Lewis , and M. R. Conte . 2021. “The La‐Related Proteins: Structures and Interactions of a Versatile Superfamily of RNA‐Binding Proteins.” RNA Biology 18, no. 2: 178–193. 10.1080/15476286.2019.1695712. 31752575 PMC7928045

[pce70581-bib-0023] Dong, S. , Z. Mao , Z. Yang , et al. 2024. “A Soybean Pyrroline‐5‐Carboxylate Dehydrogenase GmP5CDH1 Modulates Plant Growth and Proline Sensitivity.” Agronomy 14, no. 10: 2411. 10.3390/agronomy14102411.

[pce70581-bib-0024] Fernando, V. C. D. 2020. “Major Transcription Factor Families Involved in Salinity Stress Tolerance in Plants.” In Transcription Factors for Abiotic Stress Tolerance in Plants, edited by S.H. Wani , 99–109. Academic Press. 10.1016/B978-0-12-819334-1.00007-1.

[pce70581-bib-0025] Finan, J. D. , H. A. Leddy , and F. Guilak . 2011. “Osmotic Stress Alters Chromatin Condensation and Nucleocytoplasmic Transport.” Biochemical and Biophysical Research Communications 408, no. 2: 230–235. 10.1016/j.bbrc.2011.03.131. 21463604 PMC3104296

[pce70581-bib-0027] Ghosh, U. K. , M. N. Islam , M. N. Siddiqui , X. Cao , and M. A. R. Khan . 2022. “Proline, a Multifaceted Signalling Molecule in Plant Responses to Abiotic Stress: Understanding the Physiological Mechanisms.” Plant Biology 24, no. 2: 227–239. 10.1111/plb.13363. 34796604

[pce70581-bib-0028] Gonugunta, V. K. , B. C. Nair , R. Rajhans , G. R. Sareddy , S. S. Nair , and R. K. Vadlamudi . 2011. “Regulation of rDNA Transcription by Proto‐Oncogene PELP1.” PLoS One 6, no. 6: e21095. 10.1371/journal.pone.0021095. 21695158 PMC3113909

[pce70581-bib-0029] Gu, J. , S. Ma , Y. Zhang , D. Wang , S. Cao , and Z. Y. Wang . 2020. “Genome‐Wide Identification of Cassava Serine/Arginine‐Rich Proteins: Insights Into Alternative Splicing of Pre‐mRNAs and Response to Abiotic Stress.” Plant and Cell Physiology 61, no. 1: 178–191. 10.1093/pcp/pcz190. 31596482

[pce70581-bib-0030] Honkala, A. T. , D. Tailor , and S. V. Malhotra . 2020. “Guanylate‐Binding Protein 1: An Emerging Target in Inflammation and Cancer.” Frontiers in Immunology 10: 3139. 10.3389/fimmu.2019.03139. 32117203 PMC7025589

[pce70581-bib-0031] Horie, T. , M. Sugawara , T. Okada , et al. 2011. “Rice Sodium‐Insensitive Potassium Transporter, OsHAK5, Confers Increased Salt Tolerance in Tobacco BY2 Cells.” Journal of Bioscience and Bioengineering 111, no. 3: 346–356. 10.1016/j.jbiosc.2010.10.014. 21084222

[pce70581-bib-0032] Huang, S. , S. Zhu , P. Kumar , and J. D. MacMicking . 2021. “A Phase‐Separated Nuclear GBPL Circuit Controls Immunity in Plants.” Nature 594, no. 7863: 424–429. 10.1038/s41586-021-03572-6. 34040255 PMC8478157

[pce70581-bib-0033] Huang, T.‐L. , L.‐Y. Huang , S.‐F. Fu , N.‐N. Trinh , and H.‐J. Huang . 2014. “Genomic Profiling of Rice Roots With Short‐ and Long‐Term Chromium Stress.” Plant Molecular Biology 86, no. 1: 157–170. 10.1007/s11103-014-0219-4. 25056418

[pce70581-bib-0034] Ingvardsen, C. , and B. Veierskov . 2001. “Ubiquitin‐ and Proteasome‐Dependent Proteolysis in Plants.” Physiologia Plantarum 112, no. 4: 451–459. 10.1034/j.1399-3054.2001.1120401.x. 11473704

[pce70581-bib-0135] Johnson, G. , and L. A. Schaal . 1957. “Accumulation of Phenolic Substances and Ascorbic Acid in Potato Tuber Tissue Upon Injury and Their Possible Role in Disease Resistance.” American Potato Journal 34: 200–209. 10.1007/BF02854948.

[pce70581-bib-0035] Kafri, M. , E. Metzl‐Raz , G. Jona , and N. Barkai . 2016. “The Cost of Protein Production.” Cell Reports 14, no. 1: 22–31. 10.1016/j.celrep.2015.12.015. 26725116 PMC4709330

[pce70581-bib-0037] Kawa, D. , and C. Testerink . 2017. “Regulation of mRNA Decay in Plant Responses to Salt and Osmotic Stress.” Cellular and Molecular Life Sciences 74, no. 7: 1165–1176. 10.1007/s00018-016-2376-x. 27677492 PMC5346435

[pce70581-bib-0038] Kesari, R. , J. R. Lasky , J. G. Villamor , et al. 2012. “Intron‐Mediated Alternative Splicing of *Arabidopsis P5CS1* and Its Association With Natural Variation in Proline and Climate Adaptation.” Proceedings of the National Academy of Sciences 109, no. 23: 9197–9202. 10.1073/pnas.1203433109. PMC3384178 22615385

[pce70581-bib-0039] Kim, J. Y. , S. J. Park , B. Jang , et al. 2007. “Functional Characterization of a Glycine‐Rich RNA‐Binding Protein 2 in *Arabidopsis thaliana* Under Abiotic Stress Conditions.” Plant Journal 50, no. 3: 439–451. 10.1111/j.1365-313X.2007.03057.x. 17376161

[pce70581-bib-0040] Kim, Y. S. , S. I. Park , and J. J. Kim , et al. 2022. “Over‐Expression of Dehydroascorbate Reductase Improves Salt Tolerance, Environmental Adaptability and Productivity in *Oryza sativa* .” Antioxidants (Basel, Switzerland) 11, no. 6: 1077. 10.3390/antiox11061077. 35739975 PMC9220092

[pce70581-bib-0041] Komili, S. , N. G. Farny , F. P. Roth , and P. A. Silver . 2007. “Functional Specificity Among Ribosomal Proteins Regulates Gene Expression.” Cell 131, no. 3: 557–571. 10.1016/j.cell.2007.08.037. 17981122 PMC2443060

[pce70581-bib-0042] Kovalchuk, I. , V. Abramov , I. Pogribny , and O. Kovalchuk . 2004. “Molecular Aspects of Plant Adaptation to Life in the Chernobyl Zone.” Plant Physiology 135, no. 1: 357–363. 10.1104/pp.104.040477. 15133154 PMC429389

[pce70581-bib-0043] Krasensky, J. , and C. Jonak . 2012. “Drought, Salt, and Temperature Stress‐Induced Metabolic Rearrangements and Regulatory Networks.” Journal of Experimental Botany 63, no. 4: 1593–1608. 10.1093/jxb/err460. 22291134 PMC4359903

[pce70581-bib-0044] Król, A. , and S. Weidner . 2017. “Changes in the Proteome of Grapevine Leaves (*Vitis vinifera* L.) During Long‐Term Drought Stress.” Journal of Plant Physiology 211: 114–126. 10.1016/j.jplph.2016.11.016. 28178572

[pce70581-bib-0045] Kumar, R. , A. Mustafiz , K. K. Sahoo , et al. 2012. “Functional Screening of cDNA Library From a Salt Tolerant Rice Genotype Pokkali Identifies Mannose‐1‐Phosphate Guanyl Transferase Gene (OsMPG1) as a Key Member of Salinity Stress Response.” Plant Molecular Biology 79, no. 6: 555–568. 10.1007/s11103-012-9928-8. 22644442

[pce70581-bib-0046] Kumar, V. , J. K. Thakur , and M. Prasad . 2021. “Histone Acetylation Dynamics Regulating Plant Development and Stress Responses.” Cellular and Molecular Life Sciences 78, no. 10: 4467–4486. 10.1007/s00018-021-03794-x. 33638653 PMC11072255

[pce70581-bib-0047] Lambert, D. , and D. E. Draper . 2007. “Effects of Osmolytes on RNA Secondary and Tertiary Structure Stabilities and RNA‐Mg2+ Interactions.” Journal of Molecular Biology 370, no. 5: 993–1005. 10.1016/j.jmb.2007.03.080. 17555763 PMC1995082

[pce70581-bib-0048] Lan, P. , W. Li , and W. Schmidt . 2012. “Complementary Proteome and Transcriptome Profiling in Phosphate‐Deficient Arabidopsis Roots Reveals Multiple Levels of Gene Regulation.” Molecular & Cellular Proteomics: MCP 11, no. 11: 1156–1166. 10.1074/mcp.M112.020461. 22843991 PMC3494196

[pce70581-bib-0049] Li, Z. , B. Cheng , B. Yong , et al. 2019. “Metabolomics and Physiological Analyses Reveal β‐Sitosterol as an Important Plant Growth Regulator Inducing Tolerance to Water Stress in White Clover.” Planta 250, no. 6: 2033–2046. 10.1007/s00425-019-03277-1. 31542810

[pce70581-bib-0050] Luo, Y. , D.‐Q. Shi , P.‐F. Jia , Y. Bao , H.‐J. Li , and W.‐C. Yang . 2022. “Nucleolar Histone Deacetylases HDT1, HDT2, and HDT3 Regulate Plant Reproductive Development.” Journal of Genetics and Genomics 49, no. 1: 30–39. 10.1016/j.jgg.2021.10.002. 34699991

[pce70581-bib-0051] Lyzenga, W. J. , and S. L. Stone . 2012. “Abiotic Stress Tolerance Mediated by Protein Ubiquitination.” Journal of Experimental Botany 63, no. 2: 599–616. 10.1093/jxb/err310. 22016431

[pce70581-bib-0052] Meena, M. , K. Divyanshu , S. Kumar , et al. 2019. “Regulation of L‐Proline Biosynthesis, Signal Transduction, Transport, Accumulation and Its Vital Role in Plants During Variable Environmental Conditions.” Heliyon 5, no. 12: e02952. 10.1016/j.heliyon.2019.e02952. 31872123 PMC6909094

[pce70581-bib-0053] Miranda‐Ríos, J. A. , J. A. Ramírez‐Trujillo , D. J. Jaime‐Gallardo , et al. 2021. “ *Arabidopsis thaliana* AtTFIIB1 Gene Displays Alternative Splicing Under Different Abiotic Stresses.” Biologia Plantarum 65, no. 1: 255–264. 10.32615/bp.2021.022.

[pce70581-bib-0054] Miura, Y. 2013. “The Biological Significance of ω‐Oxidation of Fatty Acids.” Proceedings of the Japan Academy, Series B 89, no. 8: 370–382. 10.2183/pjab.89.370. PMC3832743 24126285

[pce70581-bib-0055] Nadarajah, K. K. 2020. “ROS Homeostasis in Abiotic Stress Tolerance in Plants.” International Journal of Molecular Sciences 21, no. 15: 5208. 10.3390/ijms21155208. 32717820 PMC7432042

[pce70581-bib-0056] Ohmae, E. , K. Gekko , and C. Kato . 2015. “Environmental Adaptation of Dihydrofolate Reductase From Deep‐Sea Bacteria.” Subcellular Biochemistry 72: 423–442. 10.1007/978-94-017-9918-8_21. 26174394

[pce70581-bib-0057] Pontecorvo, G. , P. Woodrow , P. Carillo , A. Fuggi , and M. G. Annunziata (2011) Salinity Stress and Salt Tolerance. In: Shanker A., Venkateswarlu B. (eds) Abiotic Stress in Plants ‐ Mechanisms and Adaptations. IntechOpen, Rijeka. 10.5772/22331.

[pce70581-bib-0058] Quilloy, F. A. , and V. P. Reyes (2024) Functional Role of RNA‐Binding Proteins in Plant Salt Tolerance. CABI Books:65–76. 10.1079/9781800623033.0005.

[pce70581-bib-0059] Richter, J. A. , J. H. Behr , A. Erban , J. Kopka , and C. Zörb . 2019. “Ion‐Dependent Metabolic Responses of *Vicia faba* L. to Salt Stress.” Plant, Cell & Environment 42, no. 1: 295–309. 10.1111/pce.13386. 29940081

[pce70581-bib-0060] Rosa Téllez, S. , R. Kanhonou , C. Castellote Bellés , R. Serrano , P. Alepuz , and R. Ros . 2020. “RNA‐Binding Proteins as Targets to Improve Salt Stress Tolerance in Crops.” Agronomy 10, no. 2: 250.

[pce70581-bib-0061] Sairam, R. K. , G. C. Srivastava , S. Agarwal , and R. C. Meena . 2005. “Differences in Antioxidant Activity in Response to Salinity Stress in Tolerant and Susceptible Wheat Genotypes.” Biologia Plantarum 49, no. 1: 85–91. 10.1007/s10535-005-5091-2.

[pce70581-bib-0062] Santos, R. B. , R. Abranches , R. Fischer , M. Sack , and T. Holland . 2016. “Putting the Spotlight Back on Plant Suspension Cultures.” Frontiers in Plant Science 7: 297. 10.3389/fpls.2016.00297. 27014320 PMC4786539

[pce70581-bib-0063] Scepankova, H. , D. Galante , E. Espinoza‐Suaréz , C. A. Pinto , L. M. Estevinho , and J. Saraiva . 2023. “High Hydrostatic Pressure in the Modulation of Enzymatic and Organocatalysis and Life Under Pressure: A Review.” Molecules 28, no. 10: 4172. 10.3390/molecules28104172. 37241913 PMC10223222

[pce70581-bib-0064] Schimanski, B. 2006. “A TFIIB‐Like Protein Is Indispensable for Spliced Leader RNA Gene Transcription in *Trypanosoma brucei* .” Nucleic Acids Research 34, no. 6: 1676–1684. 10.1093/nar/gkl090. 16554554 PMC1409817

[pce70581-bib-0065] Schwachtje, J. , S. J. Whitcomb , A. A. P. Firmino , E. Zuther , D. K. Hincha , and J. Kopka . 2019. “Induced, Imprinted, and Primed Responses to Changing Environments: Does Metabolism Store and Process Information?” Frontiers in Plant Science 10: 106. 10.3389/fpls.2019.00106. 30815006 PMC6381073

[pce70581-bib-0066] Schwinefus, J. J. , K. Modi , and N. Baka . 2017. “Temperature Dependence of L‐Proline RNA Duplex Destabilization.” Biophysical Journal 112, no. 3: 68a. 10.1016/j.bpj.2016.11.410. 28737394

[pce70581-bib-0067] Shah, K. , R. G. Kumar , S. Verma , and R. S. Dubey . 2001. “Effect of Cadmium on Lipid Peroxidation, Superoxide Anion Generation and Activities of Antioxidant Enzymes in Growing Rice Seedlings.” Plant Science 161, no. 6: 1135–1144. 10.1016/S0168-9452(01)00517-9.

[pce70581-bib-0068] Sharma, A. D. , and P. Singh . 2003. “Comparative Studies on Drought‐Induced Changes in Peptidyl Prolyl Cis–Trans Isomerase Activity in Drought‐Tolerant and Susceptible Cultivars of *Sorghum bicolor* .” Current Science 84, no. 7: 911–918.

[pce70581-bib-0069] Shi, H. , S. He , X. He , S. Lu , and Z. Guo . 2019. “An Eukaryotic Elongation Factor 2 From *Medicago falcata* (MfEF2) Confers Cold Tolerance.” BMC Plant Biology 19, no. 1: 218. 10.1186/s12870-019-1826-7. 31133003 PMC6537394

[pce70581-bib-0070] Singh, M. , K. Kaur , A. Sharma , et al. 2021. “Genome‐Wide Characterization of Peptidyl‐Prolyl Cis‐Trans Isomerases in Penicillium and Their Regulation by Salt Stress in a Halotolerant *P. oxalicum* .” Scientific Reports 11, no. 1: 12292. 10.1038/s41598-021-91602-8. 34112860 PMC8192932

[pce70581-bib-0071] Siodmak, A. , F. Martinez‐Seidel , N. Rayapuram , et al. 2023. “Dynamics of Ribosome Composition and Ribosomal Protein Phosphorylation in Immune Signaling in *Arabidopsis thaliana* .” Nucleic Acids Research 51, no. 21: 11876–11892. 10.1093/nar/gkad827. 37823590 PMC10681734

[pce70581-bib-0072] Skrzypczak, T. , M. Pochylski , M. Rapp , P. Wojtaszek , and A. Kasprowicz‐Maluśki . 2025. “The Viscoelastic Properties of *Nicotiana tabacum* BY‐2 Suspension Cell Lines Adapted to High Osmolarity.” BMC Plant Biology 25, no. 1: 255. 10.1186/s12870-025-06232-3. 39994523 PMC11852555

[pce70581-bib-0073] Sun, T. , T. Pei , L. Yang , et al. 2021. “Exogenous Application of Xanthine and Uric Acid and Nucleobase‐Ascorbate Transporter MdNAT7 Expression Regulate Salinity Tolerance in Apple.” BMC Plant Biology 21, no. 1: 52. 10.1186/s12870-021-02831-y. 33468049 PMC7816448

[pce70581-bib-0074] Szuba, A. , and G. Lorenc‐Plucińska . 2018. “Field Proteomics of *Populus alba* Grown in a Heavily Modified Environment ‐ An Example of a Tannery Waste Landfill.” Science of the Total Environment 610–611: 1557–1571. 10.1016/j.scitotenv.2017.06.102. 28712470

[pce70581-bib-0075] Szuba, A. , Ł. Marczak , I. Ratajczak , A. Kasprowicz‐Maluśki , and J. Mucha . 2020. “Integrated Proteomic and Metabolomic Analyses Revealed Molecular Adjustments in Populus × canescens Colonized With the Ectomycorrhizal Fungus Paxillus involutus, Which Limited Plant Host Growth.” Environmental Microbiology 22, no. 9: 3754–3771. 10.1111/1462-2920.15146. 32608104

[pce70581-bib-0076] Szuba, A. , E. Ratajczak , A. Kasprowicz‐Maluski , and E. Pers‐Kamczyc . 2022. “Plant Responses to Harsh Conditions of Post‐Industrial Habitats.” In Green Scenarios: Mining Industry Responding to Environmental Challenges of the Anthropocene Epoch, Edited by G. D. A. Woźniak and A. M. Jagodziński . CRC Press/Balkema – Taylor & Francis Group. 10.1201/9781003271604-21.

[pce70581-bib-0077] Szuba, A. , A. Wojakowska , and G. Lorenc‐Plucińska . 2013. “An Optimized Method to Extract Poplar Leaf Proteins for Two‐Dimensional Gel Electrophoresis Guided by Analysis of Polysaccharides and Phenolic Compounds.” Electrophoresis 34, no. 22–23: 3234–3243. 10.1002/elps.201300223. 24347272

[pce70581-bib-0078] Tang, Y. , M. I. Ho , B. H. Kang , and Y. Gu . 2022. “GBPL3 Localizes to the Nuclear Pore Complex and Functionally Connects the Nuclear Basket With the Nucleoskeleton in Plants.” PLoS Biology 20, no. 10: e3001831. 10.1371/journal.pbio.3001831. 36269771 PMC9629626

[pce70581-bib-0079] Tawaraya, K. , R. Horie , S. Saito , T. Wagatsuma , K. Saito , and A. Oikawa . 2014. “Metabolite Profiling of Root Exudates of Common Bean Under Phosphorus Deficiency.” Metabolites 4, no. 3: 599–611. 10.3390/metabo4030599. 25032978 PMC4192682

[pce70581-bib-0080] Tibesigwa, D. G. , W. Zhuang , S. H. Matola , et al. 2025. “Molecular Insights Into Salt Stress Adaptation in Plants.” Plant, Cell & Environment 48, no. 7: 5604–5615. 10.1111/pce.15544. 40211820

[pce70581-bib-0081] Tu, Y.‐T. , C.‐Y. Chen , Y.‐S. Huang , et al. 2022. “HISTONE DEACETYLASE 15 and MOS4‐Associated Complex Subunits 3A/3B Coregulate Intron Retention of ABA‐Responsive Genes.” Plant Physiology 190, no. 1: 882–897. 10.1093/plphys/kiac271. 35670741 PMC9434327

[pce70581-bib-0082] Vélez‐Bermúdez, I. C. , and W. Schmidt . 2014. “The Conundrum of Discordant Protein and mRNA Expression. Are Plants Special?” Frontiers in Plant Science 5: 619. 10.3389/fpls.2014.00619. 25426129 PMC4224061

[pce70581-bib-0083] Verta, J. , and A. Jacobs . 2022. “The Role of Alternative Splicing in Adaptation and Evolution.” Trends in Ecology & Evolution 37: 299–308. 10.1016/j.tree.2021.11.010. 34920907

[pce70581-bib-0084] Wang, F. , C.‐H. Li , Y. Liu , et al. 2024. “Plant Responses to Abiotic Stress Regulated by Histone Acetylation.” Frontiers in Plant Science 15: 1404977. 10.3389/fpls.2024.1404977. 39081527 PMC11286584

[pce70581-bib-0085] Wang, J. , L. Yao , B. Li , et al. 2016. “Comparative Proteomic Analysis of Cultured Suspension Cells of the Halophyte *Halogeton glomeratus* by iTRAQ Provides Insights Into Response Mechanisms to Salt Stress.” Frontiers in Plant Science 7: 110. 10.3389/fpls.2016.00110. 26904073 PMC4746295

[pce70581-bib-0086] Wang, Y. , Z. Liu , P. Wang , et al. 2020. “A 2‐Cys Peroxiredoxin Gene From *Tamarix hispida* Improved Salt Stress Tolerance in Plants.” BMC Plant Biology 20, no. 1: 360. 10.1186/s12870-020-02562-6. 32731892 PMC7393912

[pce70581-bib-0087] Wu, X. , Y. Zhong , Q. Chen , X. Zhang , and H. Zhang . 2020. “Enhancer of mRNA Decapping Protein 4 (EDC4) Interacts With Replication Protein a (RPA) and Contributes to Cisplatin Resistance in Cervical Cancer by Alleviating DNA Damage.” Hereditas 157, no. 1: 41. 10.1186/s41065-020-00154-w. 33054858 PMC7560020

[pce70581-bib-0088] Yao, H. , G. Li , and Z. Gao , et al. 2024. “Alternative Splicing Responses to Salt Stress in *Glycyrrhiza uralensis* Revealed by Global Profiling of Transcriptome RNA‐Seq Datasets.” Frontiers in Genetics 15: 1397502. 10.3389/fgene.2024.1397502. 39045328 PMC11263197

[pce70581-bib-0089] Yu, X. , X. Meng , Y. Liu , et al. 2019. “The Chromatin Remodeler ZmCHB101 Impacts Alternative Splicing Contexts in Response to Osmotic Stress.” Plant Cell Reports 38, no. 2: 131–145. 10.1007/s00299-018-2354-x. 30443733

[pce70581-bib-0090] Zhang, H. , and U. Sonnewald . 2017. “Differences and Commonalities of Plant Responses to Single and Combined Stresses.” Plant Journal 90, no. 5: 839–855. 10.1111/tpj.13557. 28370754

[pce70581-bib-0091] Zhang, H. , Y. Zhao , and J.‐K. Zhu . 2020. “Thriving Under Stress: How Plants Balance Growth and the Stress Response.” Developmental Cell 55, no. 5: 529–543. 10.1016/j.devcel.2020.10.012. 33290694

[pce70581-bib-0092] Zhang, S. , Z. Xiao , A. Liu , et al. 2026. “Salt Stress Adaptations in Soybean Involve Alterations in Pre‐mRNA Processing.” Plant, Cell and Environment. 10.1111/pce.15515. 40176308

[pce70581-bib-0093] Zhao, H. , D. Xing , and Q. Q. Li . 2009. “Unique Features of Plant Cleavage and Polyadenylation Specificity Factor Revealed by Proteomic Studies.” Plant Physiology 151, no. 3: 1546–1556. 10.1104/pp.109.142729. 19748916 PMC2773083

[pce70581-bib-0094] Zhou, B. , J. Wang , Z. Guo , H. Tan , and X. Zhu . 2006. “A Simple Colorimetric Method for Determination of Hydrogen Peroxide in Plant Tissues.” Plant Growth Regulation 49, no. 2–3: 113–118. 10.1007/s10725-006-9000-2.

[pce70581-bib-0095] Zhou, H. , C. Wang , T. Tan , J. Cai , J. He , and H. Lin . 2018. “Patellin1 Negatively Modulates Salt Tolerance by Regulating PM Na+/H+ Antiport Activity and Cellular Redox Homeostasis in Arabidopsis.” Plant and Cell Physiology 59, no. 8: 1630–1642. 10.1093/pcp/pcy081. 29684208

[pce70581-bib-0096] Zhou, L. , M. Y. Cheung , M. W. Li , et al. 2010. “Rice Hypersensitive Induced Reaction Protein 1 (OsHIR1) Associates With Plasma Membrane and Triggers Hypersensitive Cell Death.” BMC Plant Biology 10: 290. 10.1186/1471-2229-10-290. 21192820 PMC3022912

