# Supplementary material for: Long‐Term Salt Exposure Reprograms the Nicotiana tabacum BY‐2 Suspension Proteome and Metabolome Toward Stabilization of the Core Metabolic Pathways, Protein Turnover Machinery Modifications, and Protective Metabolome Adjustments
Source: Plant Cell Environ. 2026 May 5;49(8):5849–65. doi: 10.1111/pce.70581 (PMC13353748; doi:10.1111/pce.70581)
Supplement: Supplementary file 1 — Supporting File 1 [file PCE-49-5849-s005.docx]

**Long-term salt exposure reprograms the *Nicotiana tabacum* BY-2 suspension proteome and metabolome toward stabilization of the core metabolic pathways, protein turnover machinery modifications, and protective metabolome adjustments**

Anita Rzadkiewicz, Łukasz Marczak, Aleksander Strugała, Maria Tomys, Ewelina Ratajczak, Tomasz Skrzypczak, Przemysław Wojtaszek, Anna Kasprowicz-Maluśki and Agnieszka Szuba

**Supplementary File S1**

**Detailed BY-2 cultivation protocol and** **MS medium composition**

Suspension-cultured Tobacco BY-2 cells were maintained in liquid Murashige & Skoog (MS) medium (see Table S1-1 below) and subcultured weekly by transferring 10 mL culture into 70 mL fresh medium in 300-mL Erlenmeyer flasks, incubated at 21 °C on a gyratory shaker (120 rpm, 2.5-cm displacement).

Over a period of 18 months, Tobacco BY-2 suspension-cultured cells were gradually adapted to increasing concentrations of NaCl in the culture medium. The adaptation procedure began with supplementation of 20 mM NaCl, and the concentration was subsequently elevated in a stepwise manner every 2 months until a final concentration of 190 mM NaCl was reached. The initial transfers into salt-supplemented medium resulted in a marked reduction in cell division rates and fresh weight accumulation. However, following 4–8 passages at a given concentration, cell growth and biomass production recovered to levels comparable to those of the control cultures, at which point the NaCl concentration was further increased. Ultimately, 190 mM NaCl represented the highest concentration that permitted sustained cell growth and was therefore regarded as the maximal non-lethal dose for this cell line (Skrzypczak et al. 2025). After initial gradual adaptation, both control and salt-adapted BY-2 cells were subcultured weekly in their respective media for over 15 years.

**Detailed information on growth medium composition**

Salts (Murashige & Skoog 1962) and (per liter): 30 g sucrose, 100 mg inositol, 3 mg 2,4-D, 370 mg KH2PO4 and 1 mg thiamine-HCl (Nagata et al. 1992). The final pH=5.

| **Compound** | **Concentration (mM)** |
| --- | --- |
| NH_4_NO_3_ | 20.6 |
| KNO_3_ | 18.8 |
| H_3_BO_3_ | 0.1 |
| Na_2_MoO_4_·2H_2_O | 0.001 |
| CoCl_2_·6H_2_O | 0.0001 |
| KJ | 0.005 |
| CaCl_2_·6H_2_O | 3.0 |
| MgSO_4_·7H_2_O | 1.5 |
| MnSO_4_·H_2_O | 0.1 |
| ZnSO_4_·7H_2_O | 0.03 |
| Na_2_EDTA | 0.1 |
| FeSO_4_·7H_2_O | 0.1 |

**Table S1-1** Modified Murashige and Skoog medium composition

**Literature data:**

Murashige, T. & Skoog, F. (1962). A revised medium for rapid growth and bio assays with tobacco tissue cultures. Physiologia Plantarum (Plant Physiology), 15, 473–497. DOI: 10.1111/j.1399-3054.1962.tb08052.x.

Nagata, T., Nemoto, Y. & Hasezawa, S. (1992). Tobacco BY-2 cell line as the “HeLa” cells in the cell biology of higher plants. International Review of Cytology, 132, 1–30. https://doi.org/10.1016/S0074-7696(08)62452-3

Skrzypczak T, Pochylski M, Rapp M, Wojtaszek P, Kasprowicz-Maluśki A (2025) *The viscoelastic properties of Nicotiana tabacum BY-2 suspension cell lines adapted to high osmolarity*. BMC Plant Biology 25 (1):255. doi:10.1186/s12870-025-06232-3
