# Supplementary material for: Long‐Term Salt Exposure Reprograms the Nicotiana tabacum BY‐2 Suspension Proteome and Metabolome Toward Stabilization of the Core Metabolic Pathways, Protein Turnover Machinery Modifications, and Protective Metabolome Adjustments
Source: Plant Cell Environ. 2026 May 5;49(8):5849–65. doi: 10.1111/pce.70581 (PMC13353748; doi:10.1111/pce.70581)
Supplement: Supplementary file 3 — Supporting File 3 [file PCE-49-5849-s001.docx]

**Long-term salt exposure reprograms the *Nicotiana tabacum* BY-2 suspension proteome and metabolome toward stabilization of the core metabolic pathways, protein turnover machinery modifications, and protective metabolome adjustments**

Anita Rzadkiewicz, Łukasz Marczak, Aleksander Strugała, Maria Tomys, Ewelina Ratajczak, Tomasz Skrzypczak, Przemysław Wojtaszek, Anna Kasprowicz-Maluśki and Agnieszka Szuba

**Supplementary File S3**

**Gel-dependent proteomic data**

**Methods**

The general protein profiles were analysed by standard one-dimensional electrophoresis (1DE) of 20 µg of proteins (Szuba et al. 2013) in 12% or 4-15% polyacrylamide gels, respectively. The protein profiles were visualized with CBB solution, or 1DE gels were used for western blot analysis.

Immunodetection profiles were analysed after standard wet transfer on the PVDF (polyvinylidene fluoride) membranes. Before immunodetection, membranes were blocked for 1 h in a 1% BSA (bovine serum albumin) solution in TBST (Tris-buffered saline with Tween; 10 mM Tris–HCl, pH 8.0; 150 mM NaCl; 0.05% Tween 20). Membranes were incubated overnight, at room temperature with alkaline phosphatase-conjugated antibodies: with polyclonal anti Cu/Zn superoxide dismutase 1 (cytosolic) (SOD; Agrisera; produced in rabbit; AS22 4842) at 1:1500 dilution, polyclonal anti- Ascorbate peroxidase (cytosolic) (APX; Agrisera; produced in rabbit; AS06 180) at 1:2000 dilution, polyclonal anti phosphofructokinase (PFK1-7 Agrisera; produced in rabbit; AS23 4914) at 1:5000 dilution, polyclonal anti glyceraldehyde-3-phosphate dehydrogenase (cytosolic) (GAPC1/2; Agrisera; produced in rabbit; AS15 2894) at 1:3000 dilution, and with monoclonal anti-nitrotyrosine antibody (TyrNO; Sigma Aldrich; produced in mouse; SAB4701042) at 1:1000 dilution; all in 1% BSA/TBST.

Next, membranes were incubated in the TBST solution with secondary antibodies: with anti-Rabbit IgG (Sigma-Aldrich) or with anti-Mouse Monoclonal (Sigma-Aldrich), respectively, according to the primary antibody characteristic (all diluted at 1:30,000 µl in TBST), for 2 h, and after washes, were developed in SigmaFast solution (Sigma-Aldrich). For Tyr-NO, 5 μg of nitrated BSA (Sigma-Aldrich) was used as a positive control.

The quality of protein profiles was monitored after each transfer to Immobilon P using reversible membrane staining with Ponceau S solution (Sigma-Aldrich), in accordance with the manufacturer's recommendations. All analyses were performed in triplicate.

Densitometric analysis of Western blot bands (n = 3) characteristic of a given enzyme was performed using ImageJ. Digital images of the blots were converted to grayscale, and identical rectangular regions of interest (ROIs) were applied to each band. Band intensity was quantified as integrated density values. Background signal was determined from a nearby region of the membrane and subtracted from each measurement. For each band, three densitometric measurements were performed and averaged to obtain a value representing one replicate. Results were expressed as relative density (RD, %) values, where 100% represents the average background-corrected value for the control variant on a given Western blot membrane (replicate).

**Results**

***Electrophoresis-related proteomic data: oxidative stress-related data***

Proteome 1DE profiles of adapted cells were very similar to controls, with major protein bands exhibiting similar intensity across the compared variants, revealing a lack of large shifts in the most abundant proteins caused by NaCl adaptation (Figure S3-1a and Figure S3-2a). Analysis of the level of nitrotyrosines (considered as a stress-fingerprint; Corpas et al. 2013) revealed a lack of significant oxidative stress imprint on BY2 proteome; except for two bands, the majority of remaining proteins were characterized with a relatively stable level of Tyr-NO (Figure S3-1b). In agreement with this result, the abundance of selected antioxidative enzymes did not differ massively between controls and NaCl-exposed BY-2 suspensions (Figure S3-1c and d).


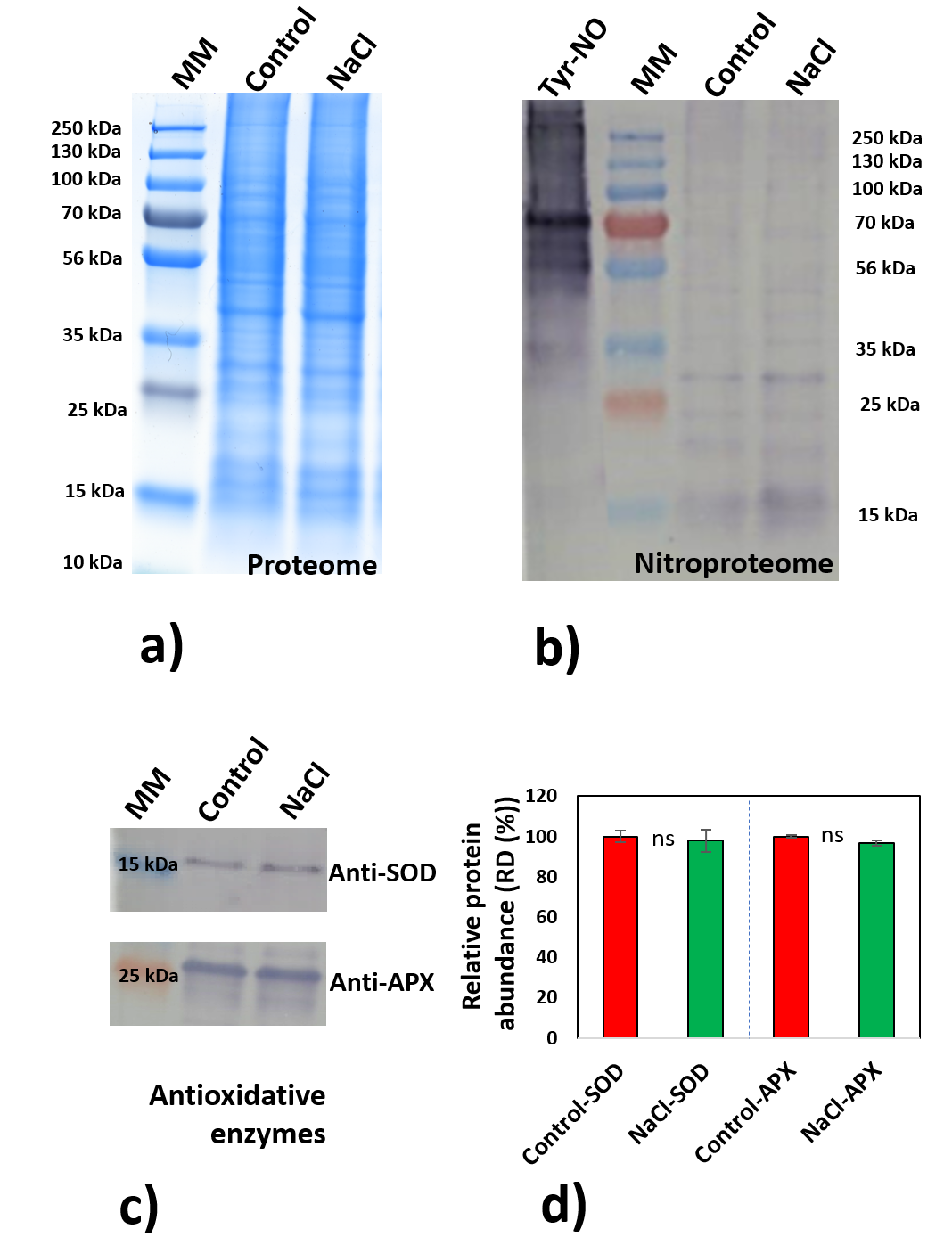


**Figure S3-1** Electrophoresis data on oxidative stress-related enzymes. Representative protein profile (1DE): 20 µg of protein extract separated in 12% PAGE (**a**). Representative nitrotyrosine levels in analysed proteins; results of immunodetection against monoclonal anti-TyrNO antibody (Sigma Aldrich). (**b**). Representative results of immunodetection of selected anti-oxidative enzymes. Standard Western blot analysis presenting the abundance of superoxide dismutase (SOD) and apoplastic peroxidase (APX) in analysed protein extracts (**c**). Densitometric analysis of Western blot bands characteristic of a given enzyme (**d**); the grey max RD (%) values are expressed as the means ± SEs (n=3). Significant differences (p ≤ 0.05) were assessed according to a t-test; ns – not significant. All experiments were performed in triplicate. Control – protein extracted from the control BY-2 cell line; NaCl – extracts originated from the salt-adapted suspension line. MM – molecular mass marker; Tyr-NO – nitrated albumin (Sigma Aldrich; Tyr-NO positive control).

The abundance of selected glycolytic enzymes, phosphofructokinase (PFK1-7) and cytosolic glyceraldehyde-3-phosphate dehydrogenase (GAPC), did not differ massively between controls and NaCl-exposed BY-2 suspensions (Figure S3-2 b and c).


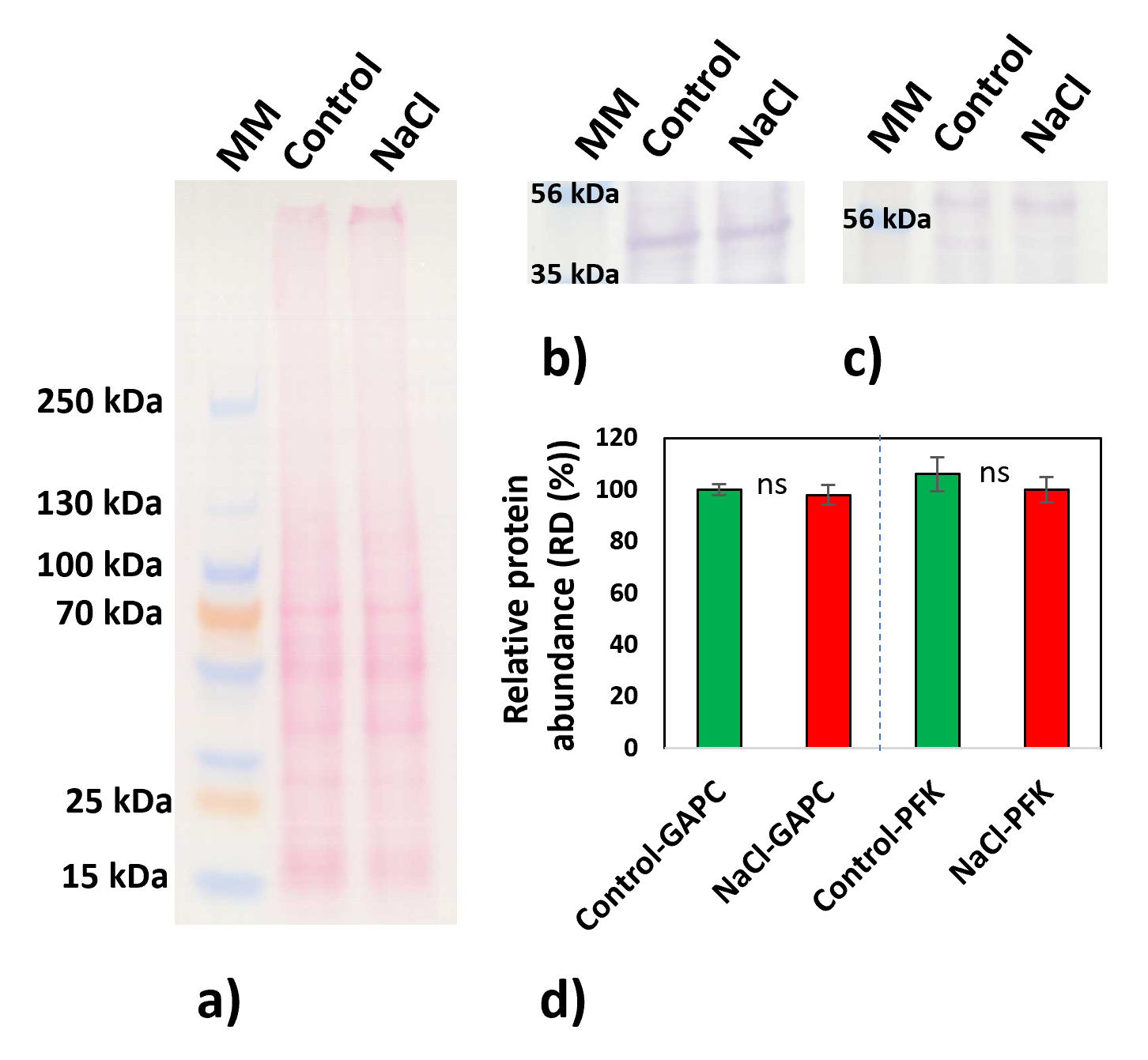


**Figure S3-2** Electrophoresis data on selected glycolytic enzymes. Representative protein profile (1DE): 20 µg of protein extract separated in 4-15% PAGE, transferred to Immobilon P membrane, and stained with Ponsou S solution (**a**). Representative results of standard Western blot analysis presenting the abundance of cytosolic Glyceraldehyde-3-phosphate dehydrogenase (b) and Phosphofructokinase 1-7 (**c**) in analysed protein extracts. All experiments were performed in triplicate. Densitometric analysis of Western blot bands characteristic of a given enzyme (**d**); the grey max RD (%) values are expressed as the means ± SEs (n=3). Significant differences (p ≤ 0.05) were assessed according to a t-test; ns – not significant. Control – protein extracted from the control BY-2 cell line; NaCl – extracts originated from the salt-adapted suspension line. MM – molecular mass marker. Arrows: bars characteristic of a given enzyme, according to the manufacturer’s instructions, which were used for densitometric analysis.

Raw, unprocessed images for the immunodetection of antioxidant enzymes with bars characteristic of a given enzyme (Figure S3-1 and S3-2), according to the manufacturer’s instructions, marked with arrows, are shown below (Figure S3-3).


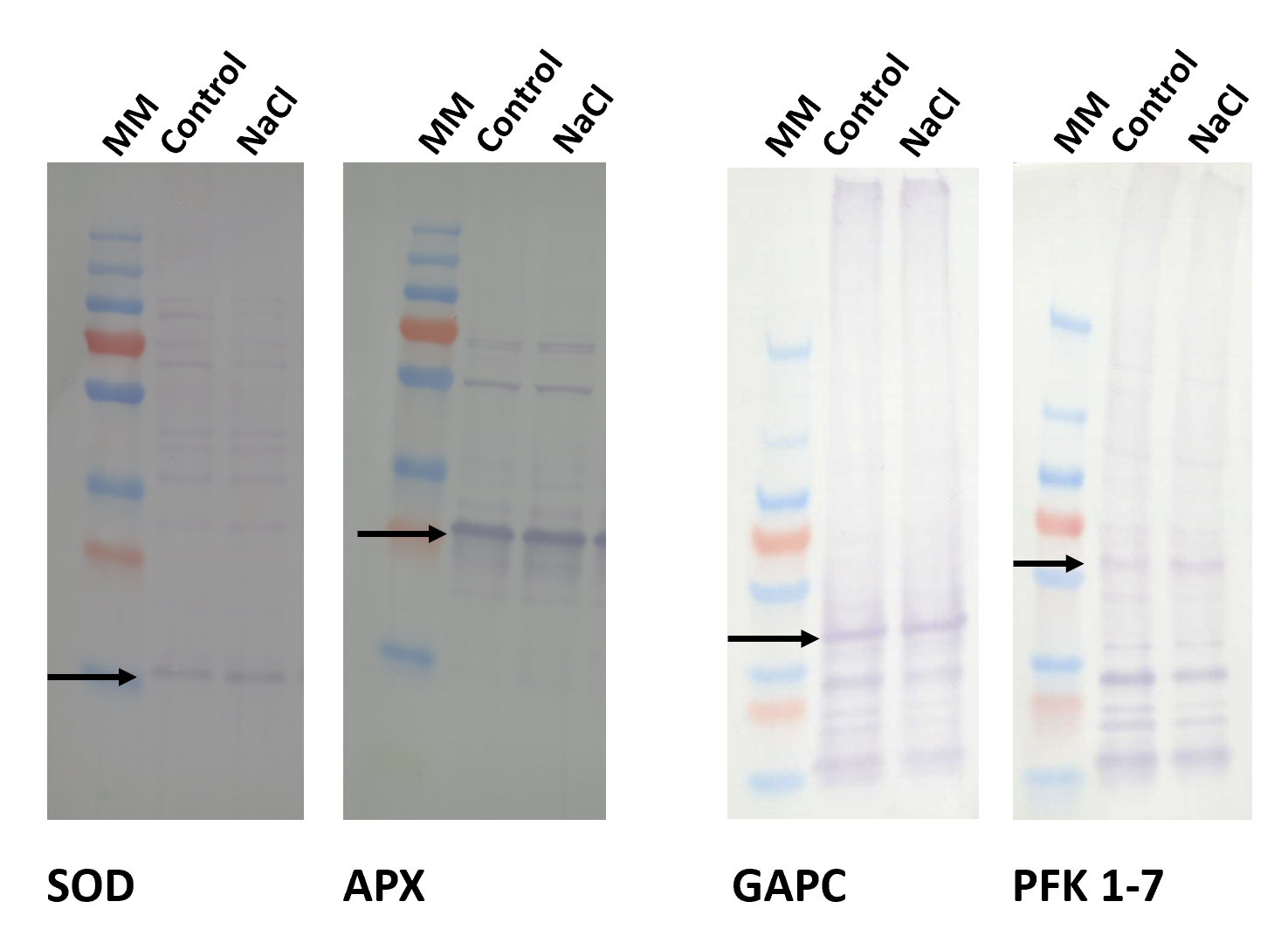


**Figure S3-3**. Representative unprocessed immunodetection images of analyzed enzymes.

**Literature data:**

Corpas FJ, Palma JM, del Río LA, Barroso JB (2013) Protein tyrosine nitration in higher plants grown under natural and stress conditions. Frontiers in Plant Science Volume 4 - 2013. doi:10.3389/fpls.2013.00029

Szuba A, Wojakowska A, Lorenc-Plucińska G (2013) An optimized method to extract poplar leaf proteins for two-dimensional gel electrophoresis guided by analysis of polysaccharides and phenolic compounds. Electrophoresis 34 (22-23):3234-3243. doi:10.1002/elps.201300223
