# Supplementary material for: Long‐Term Salt Exposure Reprograms the Nicotiana tabacum BY‐2 Suspension Proteome and Metabolome Toward Stabilization of the Core Metabolic Pathways, Protein Turnover Machinery Modifications, and Protective Metabolome Adjustments
Source: Plant Cell Environ. 2026 May 5;49(8):5849–65. doi: 10.1111/pce.70581 (PMC13353748; doi:10.1111/pce.70581)
Supplement: Supplementary file 5 — Supporting File 5 [file PCE-49-5849-s002.docx]

**Long-term salt exposure reprograms the *Nicotiana tabacum* BY-2 suspension proteome and metabolome toward stabilization of the core metabolic pathways, protein turnover machinery modifications, and protective metabolome adjustments**

Anita Rzadkiewicz, Łukasz Marczak, Aleksander Strugała, Maria Tomys, Ewelina Ratajczak, Tomasz Skrzypczak, Przemysław Wojtaszek, Anna Kasprowicz-Maluśki and Agnieszka Szuba

**Supplementary File S5**

**Analysis of mRNA levels of selected glycolytic enzymes**

**Method**

As part of the analysis of the selected glycolytic proteins in BY-2 suspension cells from the control group and in cells adapted to NaCl, the expression levels of transcripts encoding selected glycolytic enzymes were additionally analysed (Table S5-1; the same enzymes that were analysed using Western Blot and parallel reaction monitoring (PRM) techniques; details are provided in Supplementary Files S3 and S4).

The total RNA from control BY-2 cells and 190 mM NaCl-adapted BY-2 cells was extracted using GeneJET Plant RNA Purification Mini Kit (Thermo Scientific), including DNase treatment according to the manufacturer's protocol, with three biological replicates per variant. The RNA Nano 6000 Assay Kit, used with the Bioanalyzer 2100 system (Agilent Technologies, CA, USA), was employed to assess RNA integrity. Reverse transcription was performed using 0.5 μg total RNA and a SuperscriptIII kit (Invitrogen). Quantitative real-time PCR (RT–qPCR) analyses were performed with Maxima SYBR Green Rox (Thermo Fisher Scientific), with a final primer concentration of 0.5 μM on a QuantStudio 6 Flex instrument (Applied Biosystems). The sequences of the primers used in the analysis are presented in Table S5-2. All experiments were conducted in biological and technical triplicate. The Ct values were normalized against those of the internal control (UBQ and AP2M). Fold differences in expression levels were calculated according to the 2^−ΔΔCt^ method. To analyze the significance of differences between the control and adapted line, the Student's t-test was used to check the significance of differences between the control and adapted line.

| **Name** | **Gene_ID** | **Gene_Symbol** | **Gene Description** |
| --- | --- | --- | --- |
| PPFK3-like | 107775098 | LOC107775098 | ATP-dependent 6-phosphofructokinase 3-like |
| GAPDC | 107817426 | LOC107817426 | glyceraldehyde-3-phosphate dehydrogenase, cytosolic; |
| UBQ | 107828598 | LOC107828598 | polyubiquitin (LOC107828598) |
| AP2M | 107767042 | LOC107767042 | AP-2 complex subunit mu (LOC107767042) |

Table S5-1. Description of analysed genes

| **Primer name** | **Sequence (5’>3’)** |
| --- | --- |
| PPFK_107775098_Fwd | GATAGCCGAAGGAGCAGGAC |
| PPFK_107775098_Rev | AGCTGAGGCAGACCCAAAAA |
| GAPDC_107817426_Fwd | ACCGTAAGACTAGAGAAAGAAGCC |
| GAPDC_107817426_Rev | TCGGTGAATCCCAAGATGCC |
| UBQ_107828598_Fwd | CCACACTGCATTTGGTGCTC |
| UBQ_107828598_Rev | TCTGAGCTCTCCACCTCCAA |
| AP2M_107767042_Fwd | GAGTCACAGCTCAAGTCCCG |
| AP2M_107767042_Rev | AAATTCACCATCAGGCGGCA |

Table S5-2. Sequences of the primers used

**Results**

No changes were detected in the levels of the analysed transcripts between the control variant and the NaCl- adapted BY-2 cells (Fig. S5-1). Equivalent results were obtained by normalizing the data to the second housekeeping gene (AP2M).

**
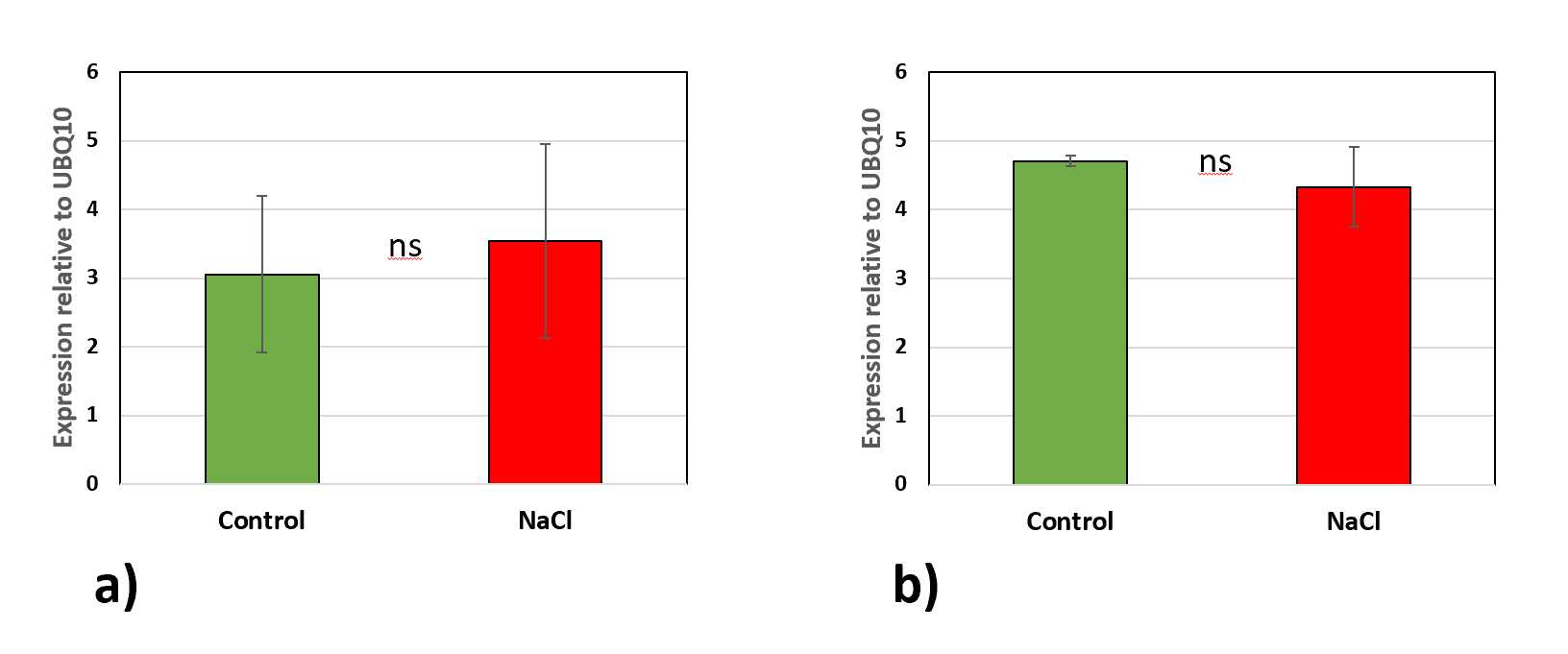
**

**Fig. S5-1**. The mRNA levels of two representative genes (see table S5-1) encoding ATP-dependent 6-phosphofructokinase (**a**), and glyceraldehyde-3-phosphate dehydrogenase (**b**), as determined by RT-qPCR expression analyses; all data for mRNA were averages from three independent experiments +/- SDs normalized to UBQ mRNA expression. Significant differences (p ≤ 0.05) were assessed according to a t-test; ns – not significant.
